# HBV capsid assembly modulators differentially modulate the assembly of wild-type and drug-resistant core protein chimeric nucleocapsids and empty capsids

**DOI:** 10.1371/journal.ppat.1013391

**Published:** 2025-08-05

**Authors:** Hui Liu, Hemraj Rimal, Jun Lyu, Liangxian Shen, Qiong Zhao, Jinhong Chang, Ju-Tao Guo

**Affiliations:** Baruch S. Blumberg Institute, Doylestown, Pennsylvania, United States of America; Pennsylvania State University College of Medicine: Penn State College of Medicine, UNITED STATES OF AMERICA

## Abstract

Multiple capsid assembly modulators (CAMs) are in clinical development for the treatment of chronic hepatitis B. The emergence of CAM-resistant HBV has resulted in the failure of CAM antiviral therapy in recent clinical trials. Because wild-type (WT) and CAM-resistant core protein (Cp) can co-assemble to form chimeric capsids, it is important to understand how CAMs modulate the assembly and disassembly of chimeric capsids and how CAM-resistant HBV variants emerge under CAM antiviral therapy. In addressing these questions, we found that in human hepatoma cells co-transfected with a serial molar ratio of WT and mutant HBV replicons expressing CAM-resistant Cp, expression of as few as 10% WT Cp conferred inhibition of nucleocapsid assembly by CAMs. However, 50% Cp with T33N substitution conferred complete resistance to the assembly of chimeric empty capsids induced by AB-506 but remained sensitive to GLS4, as determined in an *in vitro* capsid assembly assay and in transfected hepatoma cells. Moreover, the existence of approximately 50% WT Cp in chimeric nucleocapsids is required for CAMs to induce the disassembly of mature nucleocapsids and inhibit the infection of hepatocytes by HBV virions with chimeric nucleocapsids. Our results thus suggest that although disruption of nucleocapsid assembly requires only small numbers of CAM binding pockets at Cp dimer-dimer interfaces to be engaged, induction of mature nucleocapsid disassembly requires much larger numbers of CAM binding pockets to be occupied. The strong WT Cp dominance in CAM suppression of nucleocapsid assembly may slow down the emergence of CAM-resistant HBV variants under CAM therapy.

## Author summary

CAMs are a novel class of direct-acting antiviral drugs currently in clinical trials and demonstrate potent antiviral activity in patients with chronic hepatitis B. Because wild-type and CAM-resistant Cp can assemble chimeric capsids in hepatocytes, it is important to understand how CAMs interfere with capsid assembly and pgRNA packaging in cells colonized with a pool of covalently closed circular DNA (cccDNA) encoding wild-type and CAM-resistant Cp. Our results support the notion that CAM-resistant HBV variants can only be efficiently amplified in hepatocytes with more than 50% of cccDNA encoding CAM-resistant Cp. Our work also sheds new light on the mechanisms by which CAMs misdirect capsid assembly, induce the disassembly of mature nucleocapsids and inhibit the secretion of HBeAg.

Hepatitis B virus (HBV) chronically infects approximately 254 million people worldwide and causes more than 1.2 million deaths annually, due to cirrhosis and hepatocellular carcinoma (HCC) [1]. Currently available medications for chronic hepatitis B (CHB) include seven nucleos(t)ide analogue (NA) HBV DNA polymerase inhibitors and pegylated alpha-interferon (Peg-IFN-α) that modulates host antiviral immune responses [2–4]. While the NA therapy can efficiently suppress HBV replication and prevent liver disease progression in the majority of treated patients [5,6], it rarely results in the functional cure of CHB, defined as the sustained loss of serum HBV surface antigen (HBsAg) after the termination of antiviral therapy [7,8]. On the contrary, Peg-IFN-α therapy can induce functional cure in approximately 5% of CHB patients treated, but the low therapeutic efficacy and severe side-effects limit its use [9,10]. Apparently, novel antiviral agents targeting other viral components and replication steps as well as immune modulators that can more efficiently activate antiviral immune responses against HBV in CHB patients are needed for the functional cure of chronic HBV infection [11–13].

HBV replicates its genomic DNA by packaging viral pre-genomic RNA (pgRNA) and DNA polymerase (pol) into nucleocapsid where the pgRNA is reverse transcribed into a partially double-stranded, relaxed circular DNA (rcDNA) [14,15]. HBV core (capsid) protein (Cp) is a 183-amino acid (aa) polypeptide containing a N-terminal assembly domain (NTD, aa 1–140) and an arginine-rich C-terminal domain (CTD, aa150–183), linked by a 9-residue hinge. The assembly domain has five α helices connected by loops. The hydrophobic interaction between α3 and α4 helices of two Cp monomers drives the formation of a four-helix bundle at their interface and results in the formation of Cp dimers that serve as the building block of capsids [16–18]. The assembly of 120 or 90 Cp dimers into a T = 4 or T = 3 icosahedral capsid is primarily driven by the hydrophobic interaction between the Cp dimer-dimer interfaces [19]. Since the late 1990s, many chemotypes of small molecules have been discovered to inhibit the packaging of pgRNA-pol complex to form nucleocapsids and consequentially prevent viral genome replication [reviewed in [20,21]]. Structure biology and biophysics studies revealed that those compounds bind to a hydrophobic pocket, *i.e.*, HAP pocket, between Cp dimer-dimer interface to misdirect the assembly of Cp dimers into empty capsids or aberrant structures [22–26]. Those antiviral compounds are recently designated as capsid assembly modulator-empty (CAM-E) and capsid assembly modulator-aberrant (CAM-A), respectively [27]. Nevertheless, both CAM-A and CAM-E are inhibitors of pgRNA-containing nucleocapsid assembly [28,29]. Consistent with the important roles of Cp dimer-dimer interface interaction in capsid/nucleocapsid assembly and antiviral action of CAMs, we and others have demonstrated that many single amino acid substitutions of HBV Cp residues at the Cp dimer-dimer interface alter capsid assembly, pgRNA packaging, viral DNA synthesis and/or infectious virion production [30–35]. Moreover, some of those Cp mutations do not significantly reduce viral replication fitness in hepatocytes but confer resistance to one or multiple chemotypes of CAMs [32,36–38].

**Fig 1:**
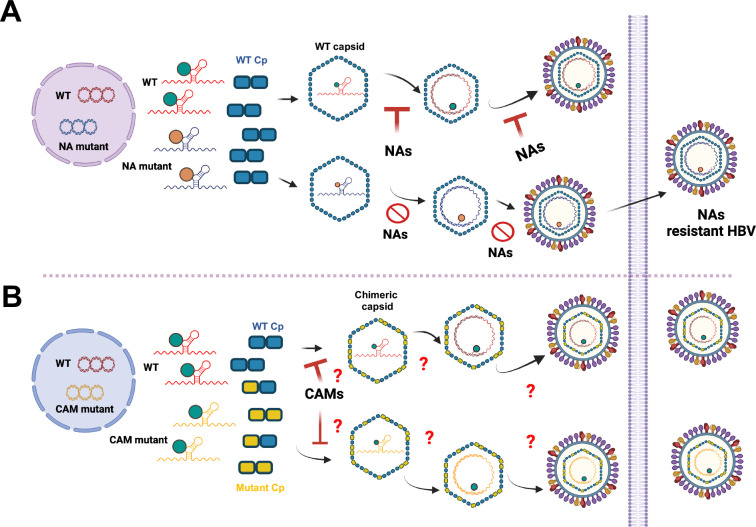
Illustration of hypothetical models for the selection of NA-resistant or CAM-resistant HBV variants under NA or CAM antiviral therapy. **(A)** Due to the cis-preferential pol and pgRNA encapsidation, pgRNA-encoding NA-resistant pol and its progeny pol protein are preferentially co-packaged into a nucleocapsid. Because reverse transcriptional HBV genome replication can only efficiently occur in the nucleocapsids packaging NA-resistant pol under NA therapy, HBV with NA-resistant genome is thus selectively amplified. **(B)** On the contrary, because WT and CAM-resistant Cp can co-assemble to form chimeric nucleocapsids, WT and CAM-resistant pgRNA might be packaged into the chimeric nucleocapsids at a similar efficiency. Therefore, amplification of HBV containing CAM-resistant genome under CAM therapy thus depends on the effect of CAMs on the assembly and disassembly of chimeric nucleocapsids in hepatocytes. The figure was created with Biorender.com.

Several CAMs have been advanced to clinical trials for the treatment of CHB and demonstrated potent antiviral efficacy [39,40]. However, like other direct-acting antiviral agents, such as nucleoside analogue HBV DNA polymerase (pol) inhibitors, emergence of CAM-resistant HBV variants resulted in the failure of CAM antiviral therapy in two recent clinical trials [37,41–44]. However, due to the unique biological functions of pol and Cp in the HBV replication cycle, the mechanism of drug-resistant virus selection under NA or CAM therapy may be different. Specifically, because the conserved prolines adjacent to the HBV pol termination codon stall ribosomes, the nascent pol is thus tethered to its template pgRNA and subsequently co-packaged into the nucleocapsid [45]. As illustrated in Fig 1A, such a *cis*-preferential pol and pgRNA packaging mechanism favor the co-assembly of a pgRNA-encoding NA-resistant pol and its progeny pol protein into a nucleocapsid. Apparently, reverse transcriptional HBV genome replication can only efficiently occur in the nucleocapsids containing NA-resistant pol under NA therapy. HBV genomes encoding NA resistance are thus selectively amplified. Like NA-resistant HBV, CAM-resistant HBV variants may also originate either from hepatocytes containing both wild-type cccDNA and cccDNA encoding CAM-resistant Cp, or hepatocytes with WT cccDNA but CAM-resistant Cp mutations generated during pgRNA transcription. Under either condition, both WT and CAM-resistant Cp will be synthesized in the same hepatocytes. Because WT and CAM-resistant Cp can co-assemble to form chimeric capsids [16,34,46], it is conceivable that either WT or CAM-resistant pgRNA can be packaged into WT and CAM-resistant Cp chimeric nucleocapsids (Fig 1B). Under this condition, the replication and secretion of WT and CAM-resistant HBV genome are equally impacted by CAM treatment. The emergence of CAM-resistant HBV variants will thus depend on the effects of CAM on the assembly and disassembly of chimeric nucleocapsids in infected hepatocytes, which have been thoroughly examined in this study.

## Results

### CAMs inhibit WT-Cp and CAM-resistant Cp chimeric HBV nucleocapsid assembly in a WT-Cp dominant manner

**Fig 2:**
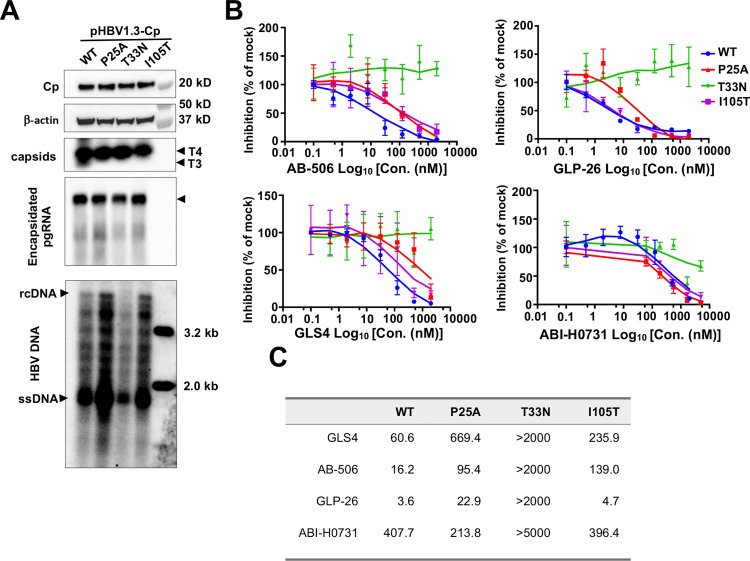
Replication fitness of CpP25A, CpT33N or CpI105T mutant HBV and their sensitivity to the representative CAM-A and CAM-E compounds in HepG2 cells. **(A)** HepG2 cells were transfected with pHBV1.3 or derived plasmid encoding the indicated mutant Cp. Cells were harvested at 72 h post-transfection. Intracellular Cp was detected by Western blot assay with HBc170A antibody. β-actin served as a loading control. Cytoplasmic capsids were detected by a native agarose gel electrophoresis-based particle gel assay. Cytoplasmic core-associated HBV DNA was detected by Southern blot hybridization. ssDNA, single-stranded DNA. rcDNA, relaxed circular DNA. Encapsidated pgRNA was detected by Northern hybridization. **(B)** HepG2 cells were transfected with pHBV1.3 or derived plasmid encoding the indicated mutant Cp. Six h post-transfection, the transfected cells were mock-treated or treated with a serial concentration of the indicated CAM. The cells were harvested at 72 h post-transfection. Cytoplasmic core-associated HBV DNA was extracted and quantified by a qPCR assay and plotted as the percentage of that in mock-treated cells. **(C)** EC_50_ values (nM) of the CAMs against WT and each of the Cp mutant HBV DNA replication are calculated from an experiment with three biological replicates using Prism GraphPad version 9.

To investigate the effects of CAMs on WT and CAM-resistant Cp chimeric HBV nucleocapsid assembly, we first characterized the replication property of WT and three mutant HBV replicons encoding Cp with a single amino acid residue substitution, P25A, T33N or I105T, that confers resistance to different CAMs in HepG2 cells [32,33]. As shown in Fig 2A, compared to WT HBV replicon, the three Cp mutations did not apparently alter the level of intracellular Cp and the amounts of total capsids. In agreement with our previous report [33], CpT33N mutation slightly compromised pgRNA packaging and HBV DNA replication, whereas CpP25A mutation supported more efficient HBV DNA replication. Antiviral assays further confirmed that CpT33N conferred a strong resistance to all the CAMs tested, but CpP25A and CpI105T mutation only conferred strong resistance to GLS4 and AB-506, respectively (Fig 2B and 2C) [32,33,36,37].

**Fig 3:**
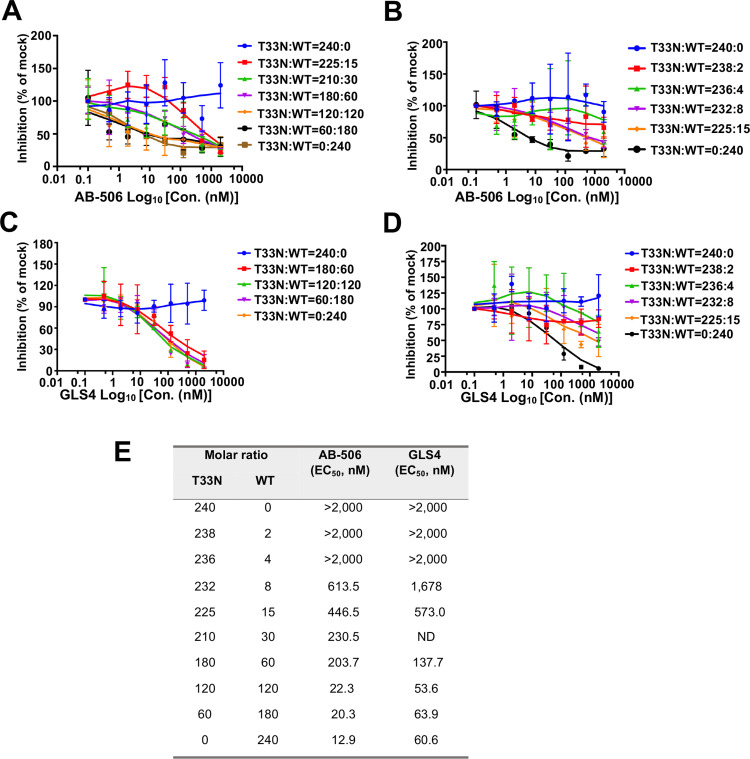
Effects of CAMs on HBV DNA replication in HepG2 cells transfected with a serial ratio of WT and CAM-resistant Cp-expressing HBV replicon plasmids. HepG2 cells were co-transfected with WT and CpT33N HBV replicon plasmids at a range of different molar ratios and treated with a serial concentration of AB-506 (panel A and B) or GLS4 (Panel C and D) for 66 **h.** The levels of cytoplasmic capsid-associated HBV DNA were determined by a qPCR assay and plotted as the percentage of that in mock-treated control cells. **(E)** EC_50_ values of the CAMs to each of the WT:CpT33N mutant replicon ratios are calculated from results shown in panel B with three biological replicates using Prism GraphPad version 9.

To determine the effect of CAMs on HBV DNA replication in cells expressing both WT and CAM-resistant Cp, HepG2 cells were co-transfected with WT HBV replicon and replicon encoding an indicated CAM-resistant Cp at a range of different molar ratios and treated with a serial concentration of an indicated CAM. The levels of HBV DNA in the cytoplasmic nucleocapsids were determined by a qPCR assay. The concentration of CAM that reduced the amounts of HBV DNA by 50% (EC_50_) were determined for each of compounds in cells transfected with each of the different WT and mutant Cp replicon molar ratios. The results presented in Fig 3 showed that compared to WT HBV replicon only transfected cells, the EC_50_ values of either GLS4 or AB-506 did not apparently change until the molar ratio of WT to CpT33N HBV replicon was below 120:120. However, CAM inhibition of HBV DNA replication can still be observed when the molar ratio of WT to CpT33N HBV replicon decreased to 8:232. Similarly, compared to WT HBV replicon only transfected cells, the EC_50_ values of GLS4 did not apparently change until the molar ratio of WT to CpP25A or CpI105T mutant HBV replicon decreased to 60:180 (S1 Fig). However, the EC_50_ values of AB-506 slightly increased when the molar ratio of WT to CpP25A or CpI105T mutant HBV replicon decreased from 240:0–60:180 (S1 Fig). These results indicate that in the presence of equal molar ratio of WT and CAM-resistant Cp, WT-Cp dominates the response to CAM inhibition of HBV DNA replication, presumably by inhibition of pgRNA-containing nucleocapsid assembly [28,30].

**Fig 4:**
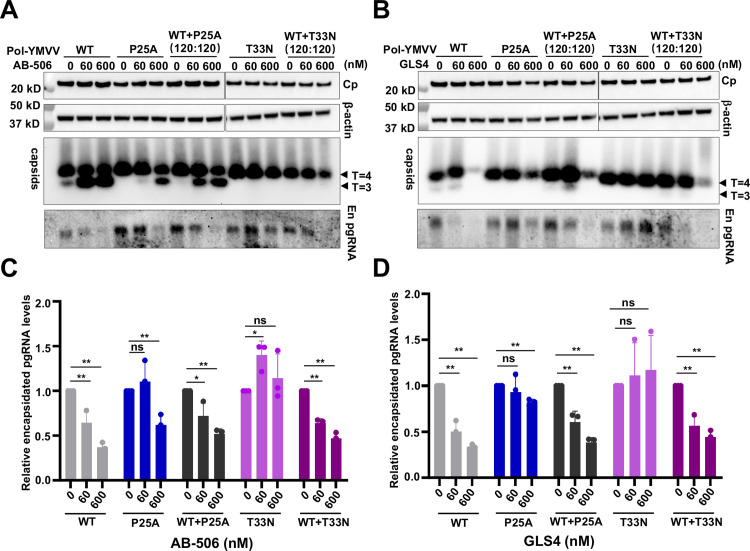
CAMs inhibit the assembly of WT and CAM-resistant Cp chimeric nucleocapsids in a wild-type Cp-dominant manner. **(A** and **B)** Huh7.5 cells were transfected with *in vitro* transcribed pgRNA/pol-YMVV, pgRNA/pol-YMVV/CpP25A and pgRNA/pol-YMVV/CpT33N, alone or in combination at 120:120 molar ratio. 3 h post-transfection, the cells were mock-treated or treated with the indicated concentrations of AB-506 or GLS4 for 12 **h.** Intracellular Cp were detected by Western blot assay with HBc170A antibody. β-actin served as a loading control. Intracellular capsids were detected by a native particle gel assay. T = 4 and T = 3 capsids are indicated. Encapsidated pgRNA was detected by Northern blot hybridization. **(C** and **D)** The band of pgRNA was quantified by ImageJ software and relative levels of encapsidated pgRNA obtained from three independent experiments were plotted. Statistical analysis was performed by unpaired t test using GraphPad Prism software. “ns” indicates no significant difference; *: *p* < 0.05; **: *p* < 0.01.

To directly examine the effects of CAMs on WT and CAM-resistant Cp chimeric nucleocapsid assembly, we took the advantage of pgRNA-launched HBV replication system recently developed by others and us to measure the effects of CAMs on pgRNA packaging in hepatocytes transfected with different molar ratios of wild-type and CAM-resistant Cp-expressing pgRNA [47,48]. To ensure the accurate measurement of encapsidated pgRNA without the interference of reverse transcriptional HBV DNA synthesis that concomitantly degrades pgRNA template in nucleocapsids, a mutation was introduced into the pgRNA to abolish the DNA polymerase activity of its encoded pol (pol-YMVV) [47]. As anticipated, GLS4 and AB-506 treatment efficiently inhibited pgRNA packaging in Huh7.5 cells transfected with wild-type pgRNA but failed to inhibit pgRNA encapsidation in cells transfected with pgRNA encoding P25A or T33N mutant Cp (Fig 4). In agreement with that observed in HBV replicon-transfected cells, both GLS4 and AB-506 efficiently inhibited pgRNA packaging in cells transfected with 1:1 molar ratio of WT:CpP25A or WT:CpT33N pgRNA. These results further support the notion that CAMs inhibit the assembly of WT and CAM-resistant Cp chimeric HBV nucleocapsids in a wild-type Cp dominant manner.

### GLS4, a prototype CAM-A, demonstrated a CAM-E-like phenotype at lower concentration

**Fig 5:**
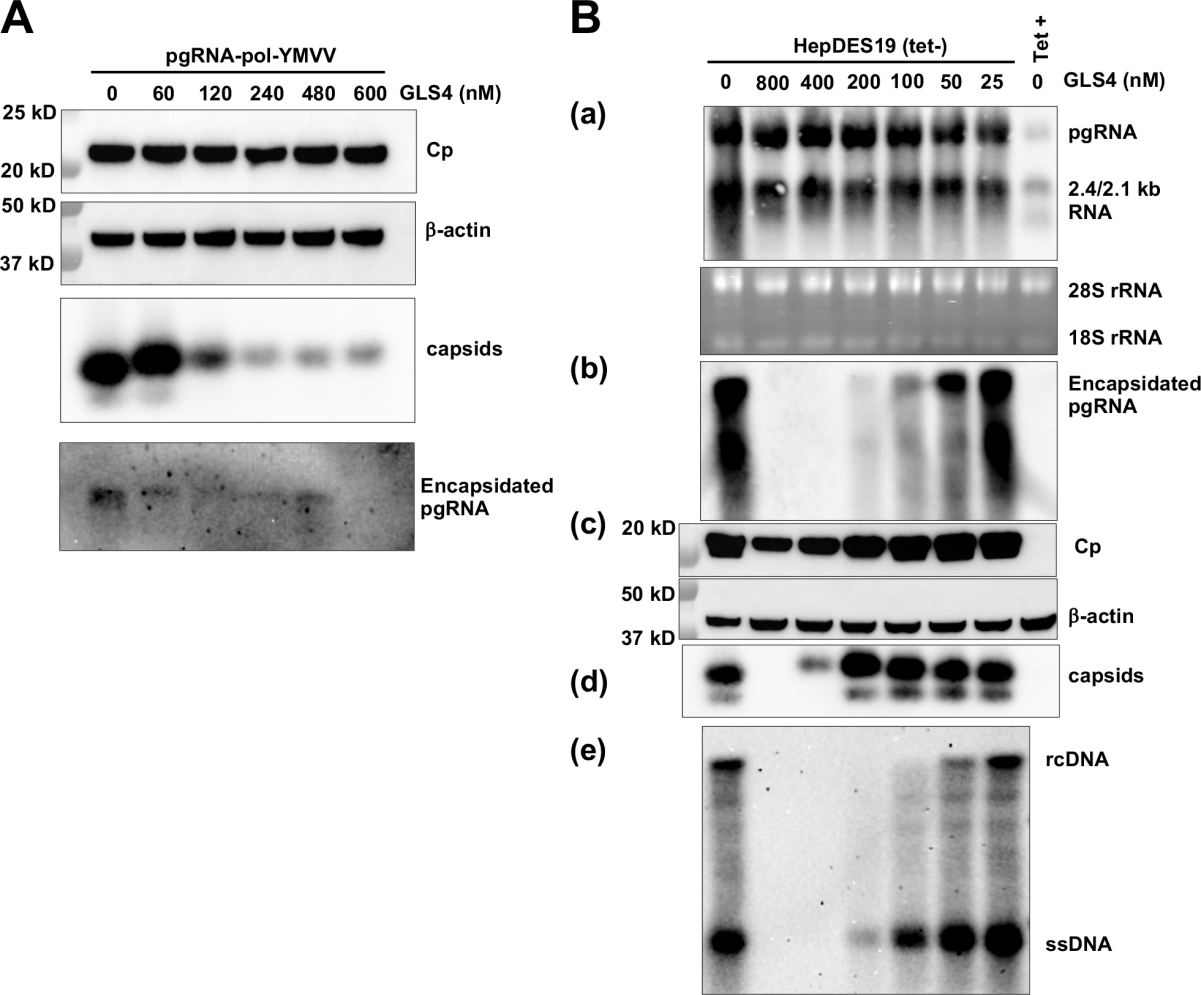
Encapsidation of pgRNA is more sensitive to GLS4 treatment than capsid assembly. **(A)** Huh7.5 cells were transfected with *in vitro* transcribed pgRNA/pol-YMVV. Three hours later, the cells were mock-treated or treated with a serial concentration of GLS4 for 12h. Intracellular Cp were detected by Western blot assay, β-actin served as a loading control. Intracellular capsids were detected by a native particle gel assay. Encapsidated pgRNA were extracted and detected by Northern blot hybridization. **(B)** HepDES19 cells were seeded into 24-well plates and cultured in the presence (Tet+) or absence (Tet-) of tetracycline. The cells cultured in the Tet- condition were treated with control solvent (DMSO) or the indicated concentration of GLS4 starting at 24 h post seeding. The cells were harvested on day 7 of treatment. Intracellular total HBV RNA (a) and encapsidated pgRNA (b) were analyzed by Northern blot hybridization. 28S and 18S rRNA served as loading control. **(c)** Core protein was detected by Western blot assay with HBc170A antibody. β-actin served as a loading control. **(d)** Intracellular capsids were analyzed by a native particle gel assay. **(e)** Intracellular core DNA was detected by Southern blot hybridization. ssDNA, single-stranded DNA. rcDNA, relaxed circular DNA.

It is well known that CAM-E misdirects the assembly of Cp dimers into T = 4 or T = 3 empty capsids whereas CAM-A induces the assembly of Cp dimers into aberrant structures that form aggregates in hepatocytes. While the cytoplasmic aggregates induced by CAM-A can be degraded *via* a STUB1-dependent autophagy pathway [49,50], the aggregates accumulated in the nuclei induce apoptosis of hepatocytes [50–54]. Interestingly, the results present in Figs 4B and 5A showed that as anticipated, treatment of pgRNA/pol-YMVV transfected Huh7.5 cells at concentration higher than 60 nM of GLS4 dramatically reduced the level of cytoplasmic HBV capsids and significantly reduced pgRNA encapsidation. However, treatment of pgRNA/pol-YMVV transfected Huh7.5 cells with 60 nM of GLS4 significantly reduced the amount of encapsidated pgRNA but did not apparently reduce the level of cytoplasmic capsids. These results suggest that this CAM-A compound worked like CAM-E at lower concentration to induce empty capsid assembly but demonstrated a CAM-A phenotype only at higher concentrations. To confirm this finding, a HepG2-derived cell line (HepDES19) supporting HBV pgRNA transcription and HBV replication in a tetracycline (tet)-off dependent manner was treated with a serial concentration of GLS4 for 6 days in the absence of tet. As anticipated, GLS4 treatment did not alter the levels of total intracellular HBV RNA (Fig 5B). In agreement with that observed in pgRNA-transfected Huh7.5 cells, treatment of HepDES19 cells with lower concentrations of GLS4 reduced the amounts of encapsidated pgRNA and cytoplasmic HBV DNA replication intermediates in a concentration-dependent manner. However, treatment of the cells with higher concentrations of GLS4 significantly reduced the levels of capsids as well as encapsidated pgRNA and HBV DNA. These results indicate that GLS4 only demonstrates its typical CAM-A-like activity, *i.e.*, inducing the assembly of aberrant non-capsid structures, only at higher concentrations. At lower concentrations, GLS4 works like CAM-E to induce the assembly of empty capsids in hepatocytes. Together with two recent studies demonstrating that CAM-A induction of HBV-infected hepatocyte apoptosis depends on high levels of Cp expression [50,53], our finding implies that the mode of action of CAMs may differ depending on the concentrations of CAM and Cp in hepatocytes.

### GLS4 and AB-506 differentially modulated chimeric empty capsid assembly

**Fig 6:**
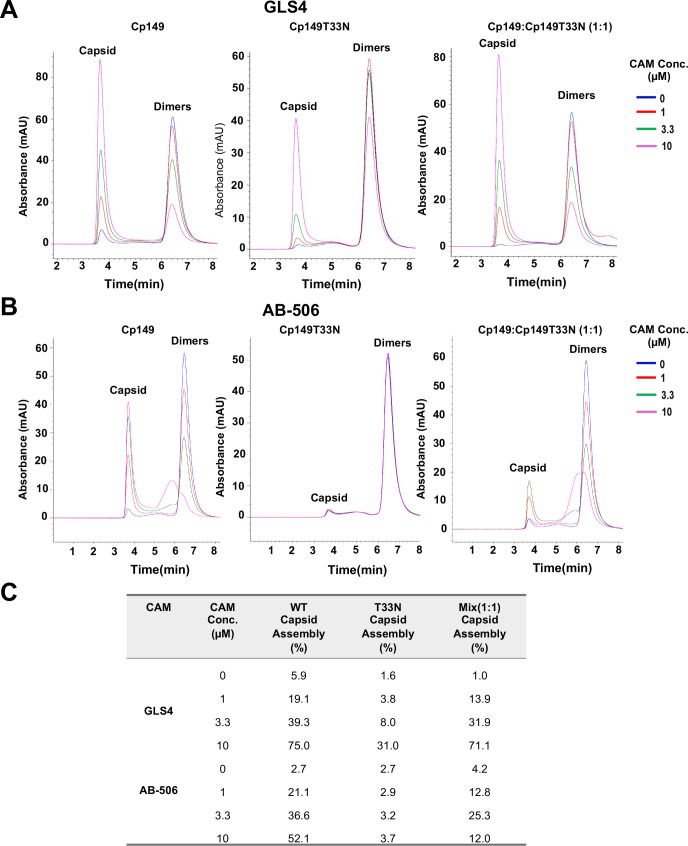
GLS4 and AB-506 differentially modulate the assembly of Cp149:Cp149T33N chimeric capsids *in vitro.* **(A** and **B)** Capsid assembly reactions were carried out with 10 μM total core protein (Cp149) in 150 mM NaCl at 37°C overnight. The assembly products with Cp149, Cp149T33N as well as Cp149 and Cp149T33N dimers at 1:1 molar ratio in the presence of indicated concentration of GLS4 or AB-506 were analyzed by size exclusion chromatography. **(C)** Capsid assembly efficiency (% capsid assembly) was calculated based on the area of Cp149 dimer’s peak from the size exclusion chromatography.

In addition to packaging pgRNA-pol complex to form nucleocapsids supporting HBV DNA replication and virion production, Cp dimers can also assemble pronominally into T = 4 empty capsids in hepatocytes. To investigate the effects of GLS4 and AB-506 on the assembly of WT and CAM-resistant Cp chimeric empty capsids, we performed *in vitro* capsid assembly assays using WT-Cp149 and T33N-Cp149 dimers expressed in and purified from *E. coli*. The light scattering analysis of Cp149 dimer assembly kinetics showed that both GLS4 and AB-506 accelerated the assembly of WT-Cp149 (but not T33N-Cp149) dimers in a CAM concentration-dependent manner. GLS4 and AB-506 accelerated the assembly of 1:1 molar ratio mixed WT-Cp and T33N-Cp149 dimers in a slightly lesser extent (S2 Fig). However, analysis of Cp dimer assembly products obtained from a prolonged (16 h) *in vitro* assembly reaction by size exclusion chromatography showed that both GLS4 and AB-506 promoted WT-Cp149 capsid assembly in a concentration-dependent manner and less efficiently induced the assembly of T33N-Cp149 capsids. Interestingly, while GLS4 induced WT-Cp149 and T33N-Cp149 (1:1) chimeric capsid assembly at an efficiency like that of WT-Cp149 capsids, AB-506 induced the assembly of the chimeric capsids with a significantly reduced efficiency as compared to WT-Cp149 capsids (Fig 6). Interestingly, AB-506 induced accumulation of partially assembled WT-Cp149 structures at higher concentrations. Taken together, the results obtained from the *in vitro* capsid assembly assay indicate while GLS4 modulates chimeric capsid assembly in a WT-Cp149-dominant manner, AB-506 modulates chimeric capsid assembly in a T33N-Cp149-dominant manner.

**Fig 7:**
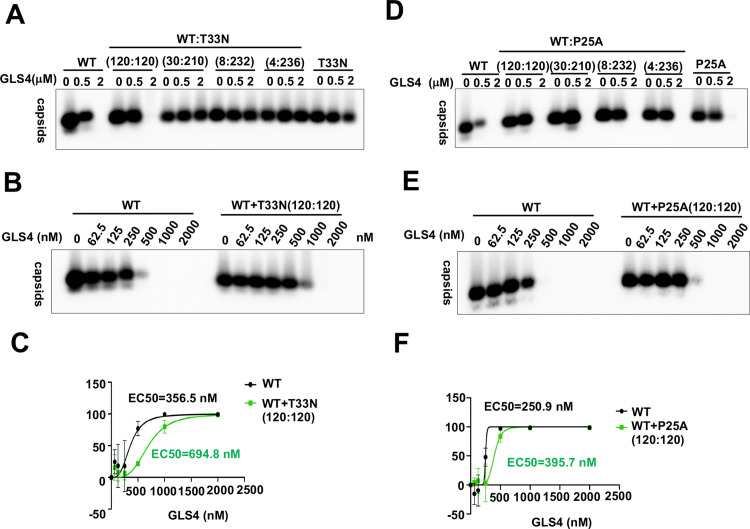
Effects of GLS4 on WT Cp, CpT33N or CpP25A chimeric empty capsid assembly in HepG2 cells. HepG2 cells were transfected with pCMV-HBc-WT, pCMV-HBc-T33N (**A** and **B**) or pCMV-HBc-P25A (**C** and **D**) alone or co-transfected with pCMV-HBc-WT and pCMV-HBc-T33N or pCMV-HBc-P25A at indicated molar ratios. Starting at 6 h post-transfection, the cells were mock-treated or treated with a serial concentration of GLS4 and harvested at 36 h post-transfection. Intracellular capsids were analyzed by native particle gel assay. The amounts of remaining capsids in **B** and **E** were determined by ImageJ software and plotted as percentage of capsid assembly inhibition, data were collected from three independent repeats. EC_50_ values were calculated using GraphPad Prism software **(C and F)**.

**Fig 8:**
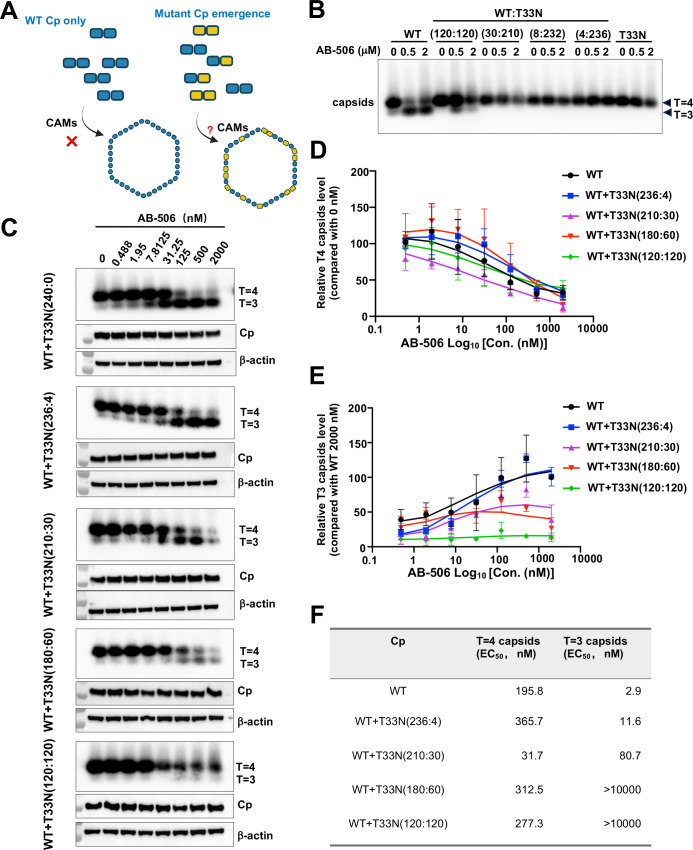
Effects of AB-506 on WT Cp and CpT33N chimeric empty capsid assembly in HepG2 cells. **(A)** Illustration of empty capsid assembly from WT Cp or WT Cp and CpT33N mixed condition examined in this experiment. The figure was created with Biorender.com. **(B** and **C)** HepG2 cells were co-transfected with pCMV-HBc-WT and pCMV-HBc-T33N at the indicated molar ratios. Starting at 6 h post-transfection, the cells were cultured with a serial concentration of AB-506 and harvested at 36 h post-transfection. Intracellular capsids were analyzed by native particle gel assay. Intracellular Cp was detected by Western blot assay. β-actin served as a loading control. (**D** and **E)** The amount of T = 4 and T = 3 capsids in each lane of panel C were quantified by ImageJ software and plotted as percentage of capsid assembly inhibition. **(F)** EC_50_ values were calculated using Prism GraphPad version 9.

We next determined the effects of GLS4 and AB-506 on chimeric capsid assembly in HepG2 cells transfected with plasmids expressing WT and CAM-resistant Cp in a titration of different molar ratios. The results presented in Fig 7A showed that GLS4 treatment resulted in a concentration-dependent reduction of WT Cp capsids, but not T33N-Cp capsids. Interestingly, GLS4 more efficiently reduced the level of WT-Cp capsids than that of chimeric capsids with WT-Cp:T33N-Cp at 120:120 ratio (Fig 7B and 7C). Further reducing the ratio of WT-Cp:T33N-Cp to 30:210 or smaller conferred complete resistance to GLS4 induced reduction of chimeric capsids. Similarly, GLS4 induced the reduction of WT-Cp capsids slightly more efficiently than the reduction of chimeric capsids with WT-Cp:P25A-Cp at 120:120 ratio (Fig 7D-7F). However, AB-506 treatment induced faster migrating T = 3 empty capsid in cells expressing WT-Cp, but not T33N-Cp. Interestingly, AB-506 induced the assembly of T = 3 capsids at the cells expressing WT-Cp and T33N-Cp at a molar ratio of 120:120 at lesser extent and failed to induce T = 3 capsids when the ratio of WT-Cp:T33N-Cp reduced to 30:210 or smaller (Fig 8B). To further characterize the dominant effect of T33N-Cp on AB-506 modulation of empty capsid assembly in hepatocytes, we performed a dose-response study of AB-506 on HepG2 cells that transfected with plasmids expressing WT-Cp and T33N-Cp at different molar ratios. The results presented in Fig 8C clearly demonstrate that AB-506 gradually induced lesser amounts of T = 3 capsids as the molar ratio of WT-Cp:T33N-Cp from 236:4 decreases to 120:120. Interestingly, the gradual decrease of T = 3 capsids under the treatment with higher concentrations of AB-506 did not result in the compensatory increase of T = 4 capsids, although the total Cp amounts did not change under all treatment conditions (Fig 8D-8F). These results indicate that higher concentrations of AB-506 treatment may misdirect the assembly of WT-Cp and T33N-Cp into non-capsid structures as observed in the *in vitro* assembly assay (Fig 6B).

In summary, the results obtained from both *in vitro* capsid assembly assay and in cells expressing WT and CAM-resistant Cp at different molar ratios consistently indicate that GLS4 modulates the assembly of WT and CAM-resistant Cp chimeric empty capsids in a WT-Cp dominant manner (Figs 6 and 7), whereas AB-506 modulates the assembly of WT- and CAM-resistant Cp chimeric empty capsids in a CAM-resistant Cp dominant fashion (Figs 6 - 8).

### CAMs induce the disassembly of mature WT-Cp:CAM-resistant Cp (1:1) chimeric nucleocapsids

**Fig 9:**
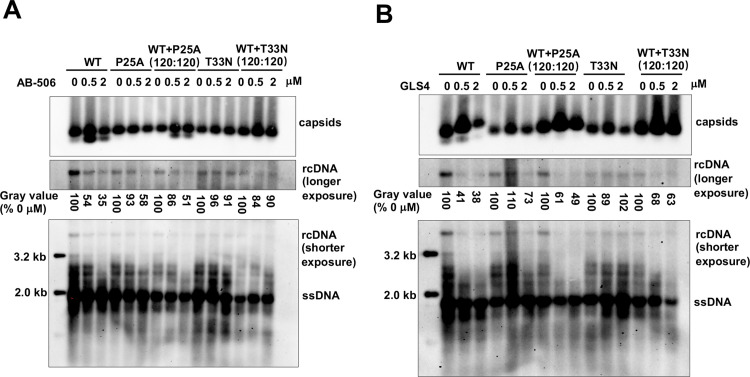
Effects of CAMs on the disassembly of WT Cp and CpP25A or CpT33N chimeric mature nucleocapsids in pgRNA transfected Huh7.5 cells. Huh7.5 cells were transfected with *in vitro* transcribed pgRNA/pol-YMVV encoding WT Cp, CpP25A, CpT33N alone or in combination at molar ratio of 120:120 (1:1). At 48 h post-transfection, the cells were mock-treated or treated with the indicated concentrations of AB-506 (**A**) or GLS4 (**B**) for six days. Intracellular capsids were detected by native particle gel assay. Cytoplasmic capsid DNA was extracted and detected by Southern blot hybridization. The gray value of rcDNA at each lane was determined by ImageJ software and expressed as the percentage of that from mock-treated cells.

In addition to modulating the assembly of empty capsids and nucleocapsids, CAMs also trigger global structural alterations of *in vitro* assembled empty capsids [23,55]. Moreover, we and others found that CAMs differentially induce the disassembly of rcDNA-containing mature nucleocapsids, which results in the premature uncoating of in-coming nucleocapsids from infecting virions and thus inhibit *de novo* cccDNA synthesis and HBV infection of hepatocytes [33,36,56–58]. Herein, we again took the advantage of the pgRNA-launched HBV replication system to examine the effects of GLS4 and AB-506 on WT and CAM-resistant Cp chimeric nucleocapsids in hepatocytes, As illustrated in (Fig 9), Huh7.5 cells were transfected with WT-, CpP25A- or CpT33N-pgRNA alone, or WT- and CpP25A-pgRNA, WT- and CpT333N-pgRNA at a molar ratio of 120:120 (1:1). At 48 h post-transfection, the cells were mock-treated or treated with the indicated concentrations of GLS4 or AB-506 for another 4 days. The cytoplasmic HBV capsids were analyzed by native agarose gel electrophoresis-based particle gel assay and nucleocapsid-associated HBV DNA were detected by Southern blot hybridization. Due to the fast degradation of transfected pgRNA, capsid/nucleocapsid assembly in the pgRNA-launch HBV replication system predominantly occur within 48 h post-pgRNA transfection [47]. As anticipated, AB-506 or GLS4 treatment of cells transfected with WT-pgRNA starting at 48 h post-transfection only slightly increased the amount of T = 3 empty (Fig 9A), upper panel) or did not reduce the amount of capsids but induced the slower migration of capsids (Fig 9B, upper panel), respectively. Therefore, the results obtained under this experimental condition mainly reflect the effects of CAMs on already assembled capsids and nucleocapsids.

As anticipated, AB-506 treatment did not induce the assembly of T = 3 empty capsid in cells transfected with CpP25A-pgRNA or CpT33N-pgRNA and GLS4 treatment failed to alter the electrophoresis mobility of capsids in cells transfected with CpP25A-pgRNA or CpT33N-pgRNA. However, GLS4 treatment slightly reduced the electrophoresis mobility of WT:CpP25A (1:1) chimeric capsids, but not WT:CpT33N (1:1) chimeric capsids, suggesting that GLS4 may fail to bind or induce the structure change of CpT33N-only capsids or WT and CpT33N (1:1) chimeric capsids. Moreover, while AB-506 or GLS4 treatment did not apparently alter the amount of single-stranded HBV DNA in all the pgRNA transfected cells, GLS4 or AB-506 treatment concentration-dependently reduced rcDNA in cells transfected with WT-pgRNA, but not CpP25A-pgRNA or CpT33N-pgRNA, suggesting that the CAMs induced the disassembly of WT, but not CAM-resistant Cp assembled rcDNA-containing mature nucleocapsids. Interestingly, treatment with either GLS4 or AB-506 also reduced amounts of rcDNA in cells co-transfected with WT-pgRNA and CpP25A-pgRNA or CpT33N-pgRNA at 1:1 molar ratio, although at a lesser extent in comparison with that in cells transfected with WT-pgRNA alone. Those results imply that approximately 50% of WT Cp is required for CAMs to efficiently induce the uncoating of mature nucleocapsids.

### CAMs inhibit the infection of HBV with WT Cp and CAM-resistant Cp (1:1) chimeric nucleocapsids

**Fig 10:**
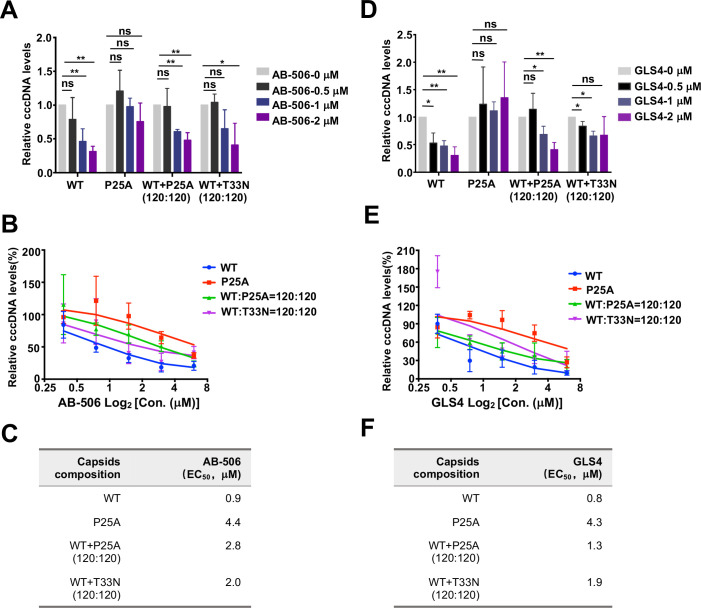
Effects of CAMs on the infection of HBV virions with WT Cp and CpP25A or CpT33N chimeric nucleocapsids. C3A^hNTCP^ cells were infected with WT HBV, CpP25A HBV, HBV virions containing WT Cp:CpP25A (120:120) or WT Cp:CpT33N (120:120) nucleocapsids. The cells were mock-treated or treated with the indicated concentration of AB-506 (**A** to **C**) or GLS4 **(D to F)**, starting at the time of infection for 6 days. Hirt DNA was extracted from the cells and cccDNA was quantified by qPCR assay with prior T5 exonuclease digestion and normalization with mitochondrial DNA (mtDNA) **(A and D)**. The relative amount of cccDNA were plotted as the ratio to that in the mock-treated cells in the dose-response curves **(B** and **E)**. EC_50_ values of AB-506 or GLS4 on cccDNA formation in the infected cultures were calculated from the dose-response curves using Prism GraphPad Version 9 **(C** and **F)**. Statistical analysis was performed by unpaired t test. ns: no significance; *: *p* < 0.05; **: *p* < 0.01.

**Fig 11:**
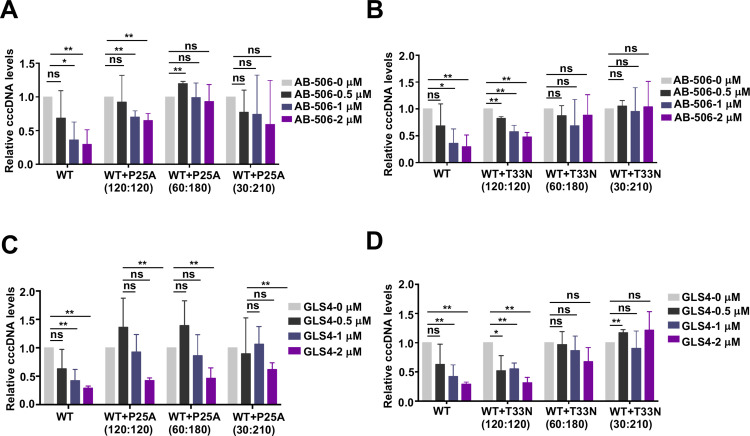
Effects of CAMs on the infection of HBV virions with WT Cp and CpP25A or CpT33N chimeric nucleocapsids. C3A^hNTCP^ cells were infected with WT HBV or HBV containing WT Cp:CpP25A (**A** and **C**) or WT Cp:CpT33N (**B** and **D**) chimeric nucleocapsids at the indicated molar ratios. The cells were mock-treated or treated with the indicated concentration of AB-506 (**A** and **B**) or GLS4 **(C and D)**, starting at the time of infection for 6 days. Hirt DNA was extracted from the cells and cccDNA was quantified by qPCR assay with prior T5 exonuclease digestion and normalization to mitochondrial DNA (mtDNA). The relative amount of cccDNA was plotted as the ratio to that in the mock-treated cells. Statistical analysis was performed by unpaired t test using Prism GraphPad Version 9. ns: no significance; *: *p* < 0.05; **: *p* < 0.01.

To test the effects of AB-506 and GLS4 on the infection of HBV with WT and CAM-resistant Cp chimeric nucleocapsids, WT HBV and HBV with WT and CAM-resistant Cp chimeric nucleocapsid were harvested from HepG2 cells transfected with WT HBV replicon or WT and CpT33N- or CpP25A-expressing replicons at the indicated molar ratios. C3A^hNTCP^ cells infected with WT or indicated chimeric HBV and treated with the indicated concentrations of AB-506 or GLS4, starting at the time of HBV infection for 6 days. HBV cccDNA in the infected cells was extracted and quantified by a cross-gap qPCR assay as previously described [33,59]. The results demonstrated that as anticipated, both AB-506 and GLS4 inhibited *de novo* cccDNA formation in WT HBV infected cells in a concentration-dependent manner and failed to inhibit cccDNA formation in Cp25A mutant HBV infected cells [33] (Fig 10). Interestingly, both AB-506 and GLS4 inhibited cccDNA formation in the cells infected with chimeric HBV with WT: CpP25A(1:1) or WT:CpT33N (1:1) nucleocapsids in a concentration-dependent manner, although at reduced activity as compared to WT HBV infection. However, AB-506 and GLS4 failed to inhibit cccDNA formation in cells infected with HBV with WT: CpP25A or WT:CpT33N chimeric nucleocapsids at 60:180 or 30:210 molar ratio (Fig 11). In agreement with findings on CAM induced rcDNA-containing nucleocapsid disassembly (Fig 9), approximately 50% of WT Cp in a chimeric nucleocapsid is required for CAMs to efficiently induce its premature disassembly and consequentially inhibit cccDNA formation in infected cells.

### GLS4 inhibition of HBeAg secretion in a wild type p17 dominant manner

**Fig 12:**
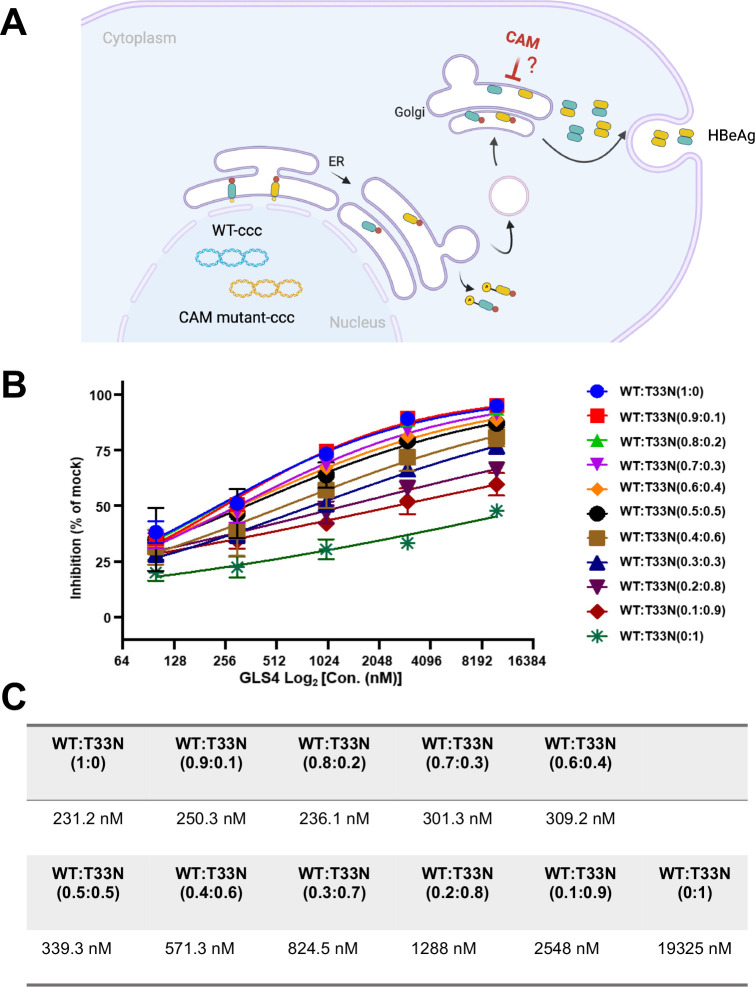
Effects of GLS4 on HBeAg secretion from HepG2 cells transfected with plasmid expressing WT p25_Δ_CTD and/or p25_Δ_CTD/T33N. (**A)** Illustration of HBeAg biogenesis pathway. p25 is co-translationally imported into ER. Removal of N-terminal signal peptide by cellular signal peptidase produces p22, which is subsequently transported to Golgi apparatus. C-terminal processing by furin protease generates p17. p17 dimers are subsequently secreted out of the cell as HBeAg. The figure was created with Biorender.com. **(B)** HepG2 cells were co-transfected with pXF3H-P25HA_Δ_CTD-WT and pXF3H-P25HA_Δ_CTD-T33N at a range of different molar ratios. Starting at 6 h post-transfection, the cells were treated with a serial concentration of GLS4. Culture media were harvested at 48 h post-transfection. HBeAg in culture media was measured by CLIA kit. (**C)** EC_50_ values of GLS4 on HBeAg secretion from HepG2 cells transfected with different ratios of the two plasmids were calculated from an experiment with three biological replicates in panel B by using Prism GraphPad version 9.

We reported previously that GLS4 inhibits the secretion of HBeAg from HepG2 cells expressing only precore protein (p25) or C-terminal truncated precore protein (p25_Δ_CTD) [33]. This finding suggests that CAM inhibition of HBeAg secretion is independent of HBV core protein expression. Interestingly, we and others also found that CAM-resistant mutations, such as CpT33N also confer resistance to CAM inhibition of HBeAg secretion [33,60]. Although p17 dimers can assemble into empty capsids in reducing condition [60,61], a particle gel assay failed to detect capsid-like structures in HepG2 cells transfected with plasmid expressing p25_Δ_CTD-WT or p25_Δ_CTD-T33N, alone or in combination at 1:1 molar ratio in the absence or presence of GLS4 treatment (S3 Fig). Based on these findings, we hypothesized that GLS4 may bind to p17 dimers to accelerate the assembly of p17 dimers into non-capsid aggregates in the Golgi apparatus or secretion vesicles, which results in the inhibition of HBeAg secretion. If this is the case, it is anticipated that co-expression of WT and T33N mutant p25_Δ_CTD will attenuate GLS4 suppression of HBeAg secretion. To test this hypothesis, HepG2 cells were co-transfected with plasmids expressing WT and T33N mutant p25_Δ_CTD at a range of different molar ratios and treated with a serial concentration of GLS4. Secreted HBeAg were quantified by CLIA assay. The results showed that like that observed in GLS4 modulation of capsid assembly in HepG2 cells (Fig 7B and 7C) or *in vitro* capsid assembly assays (Fig 6A), expression of 50% T33N p25_Δ_CTD only slightly changed the EC_50_ value of GLS4 inhibition of HBeAg secretion and existence of 10% of WT p25_Δ_CTD slightly increased the sensitivity to GLS4 suppression of HBeAg secretion (Fig 12). The results thus favor the hypothesis that GLS4 inhibition of HBeAg secretion by promoting the assembly of p17 dimer into non-capsid aggregates intracellularly.

## Discussion

Cp dimer is the building block of HBV capsid and assembly of capsid is driven by the hydrophobic interactions at Cp dimer-dimer interfaces. CAMs misdirect the assembly of Cp dimers into empty capsids or altered structures by binding to the hydrophobic interfaces between Cp dimers to accelerate the kinetics and/or alter the pathway of assembly process [62–65]. Not surprisingly, many single amino acid substitutions of HBV Cp residues at the Cp dimer-dimer interface interfere with capsid assembly, pgRNA packaging and confer resistance to CAMs [31–34]. Because wild-type and mutant Cp can assemble chimeric capsids [46], we investigated how CAMs modulate the assembly and disassembly of chimeric capsids. While the effects of CAMs on the assembly of pgRNA-containing nucleocapsids and disassembly of rcDNA-containing nucleocapsids shall provide molecular insights on the mechanism of CAM-resistant HBV variant emergence under CAM antiviral therapy, the effects of CAMs on the chimeric empty capsid assembly shed light on their mechanisms of mis-directing capsid assembly. The major findings reported herein, and their biological and medical relevance are discussed as follows.

### CAM-resistant HBV may not be selectively amplified in WT and CAM-resistant HBV co-infected hepatocytes

Amplification of HBV genome relies on the assembly of pol-pgRNA complex with Cp dimers to form nucleocapsids. Because we demonstrated previously that CAMs inhibit pgRNA encapsidation, but not reverse transcriptional viral DNA synthesis in nucleocapsids [28,56,66], the effects of CAMs on HBV DNA replication observed in HBV replicon plasmid-transfected hepatoma cells should reflect their effects on the assembly of pgRNA-containing nucleocapsids. The results presented in Figs 3 and S1 showed that the representative CAM-A (GLS4) and CAM-E (AB-506) compounds efficiently inhibited chimeric nucleocapsid assembly when the molar ratio of WT Cp and CAM-resistant Cp is 120:120. Moreover, CAMs still inhibited chimeric nucleocapsid assembly when the molar ratio of WT Cp and CAM-resistant Cp is reduced to 15:225, despite at a significantly reduced activity. The WT Cp dominance of CAM inhibition of nucleocapsid assembly was further validated in pgRNA-transfected Huh7.5 cells (Fig 4). In addition to nucleocapsid assembly, CAMs also inhibit HBV infection of hepatocytes by inducing the premature disassembly of rcDNA-containing nucleocapsids to inhibit *de novo* cccDNA synthesis [56]. We demonstrated in this report that GLS4 and AB-506 efficiently inhibited infection by HBV virions containing nucleocapsids with WT and CAM-resistant Cp at equal molar ratio (Figs 10 and 11). As illustrated in Fig 1, because WT pgRNA and pgRNA encoding CAM-resistant Cp are most likely packaged into the chimeric nucleocapsids at equal efficiency, replication of WT and CAM-resistant HBV genomes will be inhibited in hepatocytes containing both WT and CAM-resistant cccDNA and pgRNA under CAM treatment. Similarly, infection of progeny HBV virions from the co-infected hepatocytes will also be inhibited by CAMs at a similar efficacy. Because WT Cp dominates the effects of CAM inhibition of both nucleocapsid assembly and disassembly, CAM-resistant HBV genome (virus) may thus not be selectively amplified in WT- and CAM-resistant HBV coinfected hepatocytes.

### Mechanism of CAM-resistance HBV emergence under CAM therapy

The general principle of drug-resistant virus emergence under antiviral therapy is that antiviral treatment only allows the selective proliferation and spread of already-existing drug-resistant viral genomes. At the beginning of CAM therapy, CAM-resistant and WT HBV may co-colonize in some hepatocytes and CAM-resistant and WT cccDNA co-exist in the nuclei of those hepatocytes (Scenario 1) or new CAM-resistant mutation occurs during pgRNA transcription in hepatocytes containing only WT cccDNA (Scenario 2), Under both these conditions, CAM-resistant pgRNA will be packaged by both WT and CAM-resistant Cp. Another possibility is that a hepatocyte is only colonized by CAM-resistant cccDNA (Scenario 3). Apparently, the production efficiency and spread of HBV with CAM-resistant mutations under CAM therapy in Scenarios 1 and/or 2 are determined by the effects of CAMs on the assembly and disassembly of chimeric nucleocapsids. However, under the condition that a hepatocyte is only colonized by CAM-resistant cccDNA in Scenario 3, CAM-resistant HBV variants can be efficiently produced and spread under CAM therapy. Because several CAM-resistant mutations, including Cp T33N and I105T, can be detected in the serum of pretreated patients, the fast emergence of CAM resistance in the clinical trials of JNJ-56136379 and AB-506 is most likely due to the presence of hepatocytes that infected with only CAM-resistant HBV at the beginning of CAM therapy (Scenario 3). However, under the condition of Scenarios 1 and/or 2, due to the inefficient production and spread of CAM-resistant HBV variants under CAM therapy, the emergence of CAM-resistant viruses may take much longer time. An important implication of our work is that antiviral profiling of preclinical development CAM candidates against a panel of HBV variants harboring pre-existing CAM-resistant Cp mutations is critical for mitigating the risk of CAM development failure due to the fast emergence of drug-resistant HBV variants.

### Mechanistic insights on empty capsid assembly

In addition to nucleocapsids, Cp dimers also assemble into T = 4 and T = 3 empty capsids in HBV infected hepatocytes [67,68]. In fact, only approximately 10% of capsids in the infected hepatocytes are nucleocapsids [69,70]. We demonstrated previously that cellular protein phosphatase 1-catalyzed Cp C-terminal domain (CTD) dephosphorylation is required for the assembly of nucleocapsids, but not empty capsids [66,70]. However, nucleocapsids and empty capsids can be enveloped and secreted as complete (infectious) virion and incomplete genome-free virion-like particles, respectively [66,71]. Thus far, the biological function of empty capsids and genome-free virion particles remains elusive. It also remains to be determined how CAMs accelerate the Cp dimer assembly kinetics in favor of the assembly of empty capsids or aberrant structures but result in the inhibition of pgRNA encapsidation. Our results presented in this report showed that the presence of WT and T33N Cp149 at equal molar ratio did not apparently alter the yield of GLS4-induced capsid assembly *in vitro* but slightly reduced efficiency of GLS4 induced disappearance of empty capsids in HepG2 cells (Figs 6 and 7). On the contrary, the presence of 50% Cp149-T33N significantly compromised AB-506 induced assembly of capsids and resulted in the accumulation of partially assembled intermediates *in vitro* (Fig 6). While the results presented in Fig 8C demonstrate that the similar amounts of capsids are assembled under the different ratios of chimeric WT-Cp and T33N-Cp under the mock-treated conditions, AB-506 treatment differentially affects the amounts of T = 4 and T = 3 empty capsids in a concentration dependent manner. The results obtained from these studies strongly suggest that WT- and T33N-Cp do assemble to form chimeric capsids, but the increase of the ratio of T33N-Cp reduces AB-506 induction of T = 3 capsid assembly. The unique mechanism of AB-506 indued chimeric capsid assembly warranting further biophysics and structural biology studies in future.

### Mechanistic insights on HBeAg secretion

HBeAg is a secreted p17 dimer derived from the precore protein (p25) after the removal of signal peptide at its N-terminus by cellular signalase in the ER and C-terminal arginine-rich domain by furin in the Golgi apparatus [72]. Although the biological functions of p25 and its proteolytic processing and secreted products remains controversial [73], seroconversion of HBeAg to anti-HBe occurs during chronic HBV infection due to mutations in basal core promoter or precore coding region [74]. We and others reported previously that CAMs inhibit HBeAg secretion with EC_50_ values that are approximately 100-fold higher than their respective EC_50_ values of inhibiting HBV DNA replication in HBV replicon-transfected or HBV-infected hepatocytes [33,60,75]. Although one report suggests that HAP-R01 inhibited HBeAg secretion by induction of precore-derived p22 and core protein (Cp) co-assembled into non-capsid structures in hepatocytes [60], subsequent studies by our group and others demonstrated that CAM inhibition of HBeAg secretion only depends on the expression of p25, but not Cp [33,75]. Moreover, because CpT33N and a few other CAM-resistant Cp mutations also confer CAM inhibition of HBeAg secretion, and approximately 50% of intracellular p17 exists in reduced form [33], we thus hypothesized that GLS4 inhibition of HBeAg most likely occurs by promoting the reduced p17 assembly into non-capsid aggregates that cannot be properly sorted in the Golgi apparatus or secretion vesicles. The dose-dependent inhibition of T33N-mutant p17 on the secretion of HBeAg in the condition of co-expression with WT p17 in HepG2 cells strongly support this hypothesis (Fig 12). However, GLS4 treatment did not alter the intracellular p17 distribution in HepG2 cells (S4 Fig). Apparently, while our work presented herein further supports the direct engagement of GLS4 with intracellular p17 is essential for the inhibition of HBeAg secretion, further cell biology studies are required to uncover its mechanism.

Taken together, this is the first extensive study of the effects of CAMs on the assembly and disassembly of chimeric nucleocapsids and empty capsids from WT and CAM-resistant Cp. Our work uncovers the mode of action of CAMs on their multi-tropic antiviral effects and shed new light on the mechanisms of CAM-resistant HBV emergence and CAM inhibition of HBeAg secretion.

## Materials and methods

### Cell culture

Human hepatoblastoma cell line HepG2 was purchased from ATCC (HB-8065). HepDES19 is a HepG2-derived cell line supporting pgRNA transcription and HBV DNA replication in a tetracycline-off dependent manner [76]. C3A (ATCC CRL-10741) is a clonal derivative of HepG2 that was selected for strong contact inhibition of growth and high albumin production [77]. C3A^hNTCP^ is a C3A-derived cell line expressing human NTCP [78]. HepG2 and C3A^hNTCP^ were cultured in Dulbecco’s Modification of Eagle’s Medium (DMEM) (Corning) supplemented with 10% fetal bovine serum (FBS) (Gibco), 100 U/mL of penicillin, 100 μg/mL of streptomycin. Human hepatocellular carcinoma cell line Huh7.5 was obtained from Charlie M. Rice at Rockefeller University and cultured in Dulbecco modified Eagle medium (DMEM) supplemented with 10% fetal bovine serum (FBS), L-glutamine, nonessential amino acids, penicillin, and streptomycin [79].

### Chemicals and antibodies

AB-506 was provided by Arbutus Biopharma, Inc. GLS4 (Cat. NO. HY-108917), GLP-26 (Cat. NO. HY-124614) and ABI-H0731(Cat. NO. HY-109195) were purchased from MedChemExpress. Anti-HBc-170A rabbit polyclonal antibody against the C-terminal 14 amino acids (aa170–183) of HBV Cp for detecting HBV core protein by Western blot assay was generated at GenScript, Piscataway, NJ, USA and used at 1:1000 dilution [70]. Anti-β-actin (8H10D10) mouse antibody (Cat. NO. 3700) was purchased from Cell Signaling Technology and used at 1:1000 dilutions. Anti-HBcAg (1–5) mouse antibody (Cat. NO. sc-52406) used for detecting HBV capsids in particle gal assay was purchased from Santa Cruz Biotechnology and used at 1:200 dilution. Anti-HA-Tag(C29F4) rabbit monoclonal Antibody (Cat. NO. 3724S) used for detecting HA-tagged protein in Western blot assay was purchased from Cell Signaling Technology and used at 1:1000 dilution.

### Plasmids

HBV replicon pHBV1.3 as well as pCMV-HBc expressing full length HBV Cp were reported previously [32,80,81]. The pHBV1.3-derived plasmids encoding single amino acid substituted Cp and pCMV-HBc-derived plasmids expressing single amino acid substituted Cp were generated by overlapping PCR strategy [31,33]. The plasmids expressing HA-tagged p17(P25HA_DCTD_) wildtype or T33N substitution were reported previously [33]. Briefly, the backbone precore protein (p25) expression plasmids, pXF3H-p25-WT, was constructed by insertion of amplified precore coding region (nt 1816-nt 2454, genotype D, ayw) into the B*spD*I and P*st*I restricted pXF3H [82]. Mutation of the first core ATG into ATA to avoid core gene translation generates P25-WT-mATG. To generate P25HA, inserting a HA coding sequence (GTGGACATCTACCCATACGACGTTCCAGATTACGCTGGC, HA coding sequence is underlined) into the position of upstream of core in P25-WT-mATG construct. For P25HA_D_CTD, using primers (F: 5′ - CGCATC GAT ATG CAA CTT TTT CAC CTC TGC-3′ and R: 5′ - AAAACTG CAG CTACCTGCCTCGTCGTCTAACAA-3′) to amplify the P25HA, followed by ligating into p25HA after digestion the fragment and vector with B*sp*DI and P*st*I. For P25HA_D_CTD-T33N substitution, the site-directed mutagenesis was performed by using Q5 Site-Directed Mutagenesis Kit (New England Biolabs, Cat. NO. E0554), according to the manufacturer’s instruction. The plasmid of pUC57-pregenomic RNA (pgRNA) for *in vitro* transcription of full-length HBV pgRNA (a genotype D isolate, GenBank accession number U95551.1, 1820–1916 nt) is described previously [47]. Substitution of polymerase D540D541 codons (GATGAT) with GTTGTT resulted in plasmid pUC57-pgRNA/Pol-YMVV. Site-directed mutagenesis generation of desired single amino acid substitution in core protein was performed by using a commercial kit (NEB, Cat. NO. E0554) with specific primers (P25A-F: 5′- TTCTTTGCTTCAGTACGAGATCTTC-3′, P25A-R: 5′- GTCAGAAGGCAAAAACGAGAGTAAC-3′; T33N-F: 5′- TAGATAACGCCTCAGCTCTGTATC-3′, T33N-R: 5′- GAAGATCTCGTACTGAAGGAAAGAA-3′) to generate pUC57-pgRNA/PolYMVV-CpP25A and pUC57-pgRNA/PolYMVV-CpT33N. All the plasmids generated were confirmed by DNA sequencing.

### pgRNA transcription and purification

pUC57-pgRNA was linearized by AseI digestion at 37 °C for overnight and precipitated by adding a 1/10 volume of 3 M sodium acetate and 2 volumes of ethanol. The synthesis of HBV pgRNA was performed by using T7 mScript standard mRNA production System (CellScript, Cat. NO. CMSC100625) by following the manufacturer’s instruction. Purification of *in vitro* transcribed pgRNA was performed by using RNeasy mini kit (Qiagen, Cat. NO. 74014) according to the manufacturer’s instruction, including the optional on-column DNase digestion step (Qiagen, Cat. NO. 79254). Capping and polyadenylation of *in vitro* transcribed pgRNA were performed following the Cap-1 mRNA protocol described in the user’s manual and the RNA was again purified using the RNeasy mini kit without on-column DNase digestion [47].

### pgRNA transfection of Huh7.5 cells

Huh7.5 cells were seeded into 12 well plates with a density of 2.5´10^5^ cells per well. At 48 h post seeding, the cell medium was changed into 1 mL of DMEM (Corning, Cat. NO. 10–013-CV) containing 1.5% FBS and 0.1 mM nonessential amino acids (NEAA) (Gibco, Cat. NO. 11140050) per well before transfection. For each well, 0.5 µg pgRNA and 2.5 µL lipofectamine 2000 (Invitrogen, Cat. NO. 11668–019) were mixed with 250 µL Opti-MEM reduced serum medium (Gibco, Cat. NO. 31985088), followed by incubating the mixture at room temperature for 15 mins. After the mixture was added into culture medium, the transfected plates were centrifuged at 37°C for 30 min at 1000 g. Six h later, the medium was replaced with DMEM containing 3% FBS, 1% GlutaMAX (Gibco, Cat. NO. 35050079), 0.1 mM NEAA, 1´ penicillin-streptomycin (Gibco, Cat. NO. 15140122) and 1´ insulin-transferrin-selenium (Gibco, Cat. NO. 41400045). The culture media or the cells were harvested at the indicated time-points post-transfection for desired molecular analysis.

### Expression and purification of Cp149 and mutant Cp149-T33N proteins

The coding region of Cp amino acid residues 1–149 from pHBV1.3 was subcloned into pET11a^+^ vector. CpT33M mutation was introduced into the resulting Cp149-expresing plasmid by using Quikchange Mutagenesis kit (Stratagene). After confirmation of the sequence by Sanger sequencing (Quintarabio), each of the resulting plasmids was transformed into NiCo21(DE3) *Escherichia coli*. Protein expression was induced at 27°C using 1 mM isopropyl-β-d-thiogalactopyranoside when OD_600_ reached 0.8. After 24 h, the cells were harvested by centrifugation at 4000 rpm for 15 min and frozen at -80°C. For protein purification, the cells were resuspended and lysed with non-ionic detergent (B-per Complete) from Thermofisher scientific containing DNase I and Lysozyme. Further purification was done as described previously [83]. The extinction Coefficient of purified proteins at 280 nm was 60,900 M^-1 cm−1^ and it was used for quantification of dimers [84].

### In vitro capsid assembly reactions and Size-Exclusion Chromatography (SEC) analysis

Before reaction, Cp dimers were dialyzed into 20mM Tris-HCl (pH 7.4). The assembly reactions were performed with a system containing 10µM Cp149, Cp149-T33N or Cp149:Cp149-T33N (1:1), 20 mM Tris-HCl, 150 mM NaCl and indicated concentration of GLS4 or AB-506 at 37°C for 16 h [24,85]. The products of the *in vitro* capsid assembly reaction were analyzed by SEC using Superose 6 Increase 5/150 GL column (Cytiva) mounted on Agilent 1100 HPLC equipped with a diode array detector. The column was washed and equilibrated with Tris-HCl buffer (pH 7.4) [24]. Chromatographic peaks were observed at 280nm and integrated over the capsid and dimer elution times (or volumes) to determine the corresponding fraction of assembled and unassembled subunit in each reaction [86]. The integration of peaks was done using Agilent’s Chemstation software. The eluted fraction peaks were allocated either as a void (1 - 3.2 min), capsid (3.2 - 4.6 min), dimers (6–8 min), or intermediate (4.6 - 6 min) [38].

### Light scattering analysis of Cp assembly

Prior to initiating the assembly reaction, Cp149, Cp149-T33N, or a 1:1 molar ratio mixture of both dimeric protein (10 µM) was incubated with the desired concentration of GLS4 or AB-506 at room temperature for at least 10 minutes. Assembly was initiated after adding an equal volume of buffer (50 mM Tris-HCl, 600 mM NaCl, pH 7.4) to the Cp149 dimer–CAM mixture, yielding final reaction mixture containing of 50 mM Tris-HCl, 300 mM NaCl, 5 µM Cp149 or Cp149-T33N or a combination of Cp149 and Cp149-T33N at 1:1 molar ratio, and 1–10 µM GLS4 or AB-506 as indicated in S2 Fig.

### HBV stock preparation and titration

To prepare HBV virion stocks for infection assay, the culture media of HepG2 cells transfected with pHBV1.3-WT and its derived plasmids encoding mutant Cp (pHBV1.3-P25A or pHBV1.3-T33N) at different molar ratio were harvested at day 6, 9 and 12 post-transfection, respectively. Viral particles in the culture media were concentrated by 20% sucrose cushion ultracentrifugation at 27,000 rpm (Beckman, SW28) for 16 h at 4 °C. The pellet was resuspended with desired volume of Opti-MEM (Gibco, Cat. NO. 31985–070) (volume of Opti-MEM usually about 1% of culture media). Titers of HBV virions were determined by the IP-qPCR assay as described previously [33]. Briefly, thirty microliters of concentrated viral stock were mixed with 970 µL Phosphate Buffered Saline (PBS) (Corning, Cat. NO. 21–040-CMX12) and precleared by addition of 20 µL Dynabeads Protein G (Invitrogen, Cat. NO. 10004D). The mixture was rotated at 4°C for 60 min. After the removal of beads, anti-HBsAg antibody (Abcam, Cat. NO. ab9193) and anti-preS2 antibody (Abcam, Cat. NO. ab8635) were added at a ratio of 1:2 for a total of 6 µL into the pre-cleared supernatant and incubated overnight at 4 ºC. Thirty microliters of Dynabeads Protein G were then added into each sample and incubated at 4°C for 4 h. The beads were washed with PBS for 10´ 5 mins. The beads were collected and resuspended in core DNA lysis buffer (10 mM Tris-HCl, pH 8.0; 1 mM EDTA; 1% Nonidet P-40). Before extracted by phenol-chloroform, the samples were digested by DNase I (Promega, Cat. NO. M610A) at 37°C for 30 min and followed by proteinase K (200 µg/mL) digestion at 50°C for 1 h. For quantification of HBV DNA, a SYBR Green Real-time PCR method were performed by Light-Cycler 480 II Real-time PCR Detection System (Roche, Mannheim, Germany). The primers used to detect HBV viral titer is as follows: F (303–322 nt): 5′-TGGCCAAAATTCGCAGTCCC-3′, R (448–425 nt): 5′-GAAGAACCAACAAGAAGATGAGGC-3′ [87]. The serial dilutions of pHBV1.3 plasmid were used as standards of quantification.

### HBV infection of C3A^hNTCP^ cells

For C3A^hNTCP^ cells infection, cells were seeded into rat tail collagen-I-coated plates for 24 h and supplied with DMEM containing 10% FBS, then pretreated with DMEM supplemented with 3% FBS, 1% MEM NEAA (Gibco, Cat. NO. 11140) and 2% DMSO for 24 h. The cells were then infected with HBV in DMEM containing 3% FBS, 2% DMSO, 1% MEM NEAA and 4% PEG-8000 (Sigma, Cat. NO. P1458). The inoculum was removed at 16–24 h post-infection (hpi) and the cell monolayers were washed with PBS 5 times before refreshing with DMEM containing 3% FBS, 1% MEM NEAA and 2% DMSO. The cells were harvested at indicated time point.

### Extraction and detection of HBV DNA and RNA by hybridization and real time PCR assays

HBV capsid-associated (core) DNA was extracted from transfected or infected cells as described previously [76]. For extraction of encapsidated pgRNA, the cells were lysed by addition of 250 μL lysis buffer (10 mM Tris-HCl, pH 8.0; 1 mM EDTA; 1% Nonidet P-40) per well of 12-well plates and incubated in room temperature (RT) for 20 min. The cell lysates were centrifugated at 12,000 *g* for 10 min at 4°C to remove cell debris. The supernatant was mixed with 6 Units of micrococcal nuclease (New England Biolabs, Cat. NO. M0247S) and 15 μL 100 mM CaCl_2_ and incubated at 37°C for 15 min. The reaction was terminated by the addition of 6 µL of 0.5 M EDTA. Encapsidated HBV pgRNA in the reaction was extracted by adding 750 µL of Trizol LS reagent (Invitrogen, Cat. NO. 10296010) by following manufacturer’s directions. Southern blot and Northern blot detection of HBV DNA and RNA were performed with a modified digoxin (DIG) method. Briefly, for making the DIG HBV DNA probe, using pCMV-HBV [28,81] as the template with the primers (DIG-F: 5′-TTTTTCACCTCTGCCTAATCA-3′ and DIG-R: 5′-AAAAAGTTGCATGGTGCTGG-3′) to perform PCR reaction by following the PCR DIG Probe Synthesis Kit instructions (Roche, Cat. NO. 11636090910). After agarose gel electrophoresis, membrane transferring and UV-crosslinking, HBV DNA or RNA were hybridized with DIG Easy Hyb (Roche, Cat. NO.11603558001) containing DIG HBV DNA probe. After hybridization, the membrane was washed with buffer I (2 × SSC, 0.1% SDS) for 2 × 5 minutes at room temperature, then washed with buffer II (0.2 × SSC, 0.1% SDS) for 2 × 20 minutes at 60°C. The membranes were then blocked with DIG Blocking buffer (Roche, Cat. NO.11585762001) for 30 minutes, followed by probing with anti-Digoxigenin-AP (Roche, Cat. NO.11093274910) for 30 minutes. After washing with DIG washing buffer (Roche, Cat. NO. 11585762001) for 3 × 15 minutes, the membrane was incubated by CDP-Star (Roche, Cat. NO. 11759051001) for 10 minutes and the DIG signal was detected by ChemiDOC Touch Image System (BioRad). For HBV DNA quantification by Real-time PCR, a SYBR Green Real-time PCR method were performed by Light-Cycler 480 II Real-time PCR Detection System (Roche, Mannheim, Germany). The primers used to detect HBV DNA is as follows: F (303–322 nt): 5′-TGGCCAAAATTCGCAGTCCC-3′, R (448–425 nt): 5′-GAAGAACCAACAAGAAGATGAGGC-3′ [87].

### Detection of HBV cccDNA

HBV cccDNA was extracted by a modified Hirt DNA extraction method [76]. For detection of cccDNA, four-fifths of Hirt DNA extracted from a six well plate was denatured at 88°C for 8 minutes and chilled in ice for 5 min. The samples were then digested by E*coR*I at 37°C for 60 min and subjected to Southern blot detection of HBV cccDNA. The remaining one-fifth of Hirt DNA was mixed with 5 μL 2 × NEB 3 buffer (NEB, Cat. NO. B7003S), 1 μL T5 exonuclease (NEB, Cat. NO. M0663S) and nuclease-free water for a total of 10 μL and incubated at 37°C for 30 min. The reaction was terminated by incubation at 95°C for 5 min and subjected for quantification of HBV cccDNA by real time PCR. Specifically, the reaction mixture (20 μL) contained 1 μL of forward primer (10 μM) (5′-GCCTATTGATTGGAAAGTATGT-3′), 1 μL of reverse primer(5′-AGCTGAGGCGGTATCTA-3′) (10 μM), 4 μL of cccDNA template, 10 μL of 2 × mix LightCycler 480 SYBR green Master (Roche, Cat. NO. 4887352001), and 4 μL of nuclease-free water. The reaction mixture was denatured at 95°C for 5 minutes, followed by 45 cycles at 95°C for 30 seconds, 60°C for 30 seconds, and 72°C for 30 seconds, 88°C for 2 seconds [88]. A serial dilution of pHBV1.3 plasmid were used as standards of quantification [33].

### Western blot assays

For 12-well plate, cells in each well were lysed in 250 μL of 1 × LDS buffer (Invitrogen, Cat. NO. NP0007) with 2.5% 2-mercaptoethanol (Sigma) at RT for 10 min. The lysates were incubated at 100 °C for 20 min. An aliquot of 20 μL of the cell lysate was resolved in a NuPAGE 12% Bis-Tris Protein Gel (Invitrogen, Cat. NO. NP0343BOX) with NuPAGE MES SDS running buffer (Invitrogen, Cat. NO. NP0002) and transferred onto PVDF membrane (Invitrogen, Cat. NO. NP0002) by using iBlot 2 dry blotting system (Thermo fisher scientific). The membrane was blocked with 5% nonfat milk in TBST (Tris-buffered saline containing 0.1% Tween 20) at RT for 1 h and probed with a desired primary antibody. Bounded antibody was revealed either by HRP-linked anti-rabbit/mouse IgG secondary antibodies and visualized by ChemiDOC Touch Image System (BioRad).

### ELISA detection of HBeAg

HBeAg was detected by commercial HBeAg ELISA CLIA kit (Autobio, Cat. NO. CL0312–2) according to manufacturer’s instruction.

### Immunofluorescence staining

Huh7 cells were seeded on coverslips in 24-well plates and transfected with p25HA_△_CTD-WT, p25HA_△_CTD-T33N or a mixture of p25HA_△_CTD-WT and p25HA_△_CTD-T33N at a molar ratio of 1:1. At 6 h post transfection, the cells were mock-treated with control solvent (DMSO) or GLS4 (10 µM) for 48 h. The cells were fixed with 95% methanol and 5% glacial acetic acid and then washed 3 times with 1 × PBS followed by 1 h of incubation with the blocking and permeabilization buffer (5% BSA, 10% FBS, 0.3% Triton-X-100 in 1 × PBS, all (vol/vol)) at room temperature for 1 h. Thereafter, cells were incubated with an anti-HA antibody (1:100 dilution, Cat. NO. 3724S, Cell Signaling Technology, MA) in the dilution buffer (1% BSA, 1% FBS, 0.3% Triton-X-100 in 1 × PBS) at 4 °C overnight, washed for 3 times and incubated with an Alexa Fluor 488 conjugated goat anti-rabbit secondary antibody (1:1000 dilution, Cat. NO. A-11034, Invitrogen, MA). The nuclei were visualized by DAPI (1 µg/mL) in dilution buffer (1% BSA, 1% FBS, 0.3% Triton-X-100 in 1 × PBS) at room temperature for 1 h. After 3 times of washing, the cover slides with cells were then transferred onto a glass slide with 2 µl ProLong Gold antifade solution (Cat. NO. P36930, Thermo Fisher Scientific, Waltham, MA) and sealed with CoverGrip Coverslip Sealant (Cat. NO. 23005, Biotium Inc., Fremont, CA). The slides were then covered with foil and dried overnight at 4 °C and then analyzed with a confocal microscope (Nikon A1R-STED) using a 60 × objective. Scale bar: 20 μm.

### Statistical analysis

Statistical analysis was performed by unpaired student *t* test using Prism software Prism GraphPad Version 9. The value of **P* *< 0.05 was considered statistically significant. NS: no significance; *: *p < 0.05;* **: *p < 0.01;* ***: *p < 0.001*. The EC_50_ values were obtained by plotting dose response curves and using GraphPad Prism 9 with all the experiments performed in three biological replicates.

## Supporting information

S1 Fig Effects of CAMs on HBV DNA replication in HepG2 cells transfected with a serial ratio of WT and CAM-resistant Cp-expressing HBV replicon plasmids. HepG2 cells were co-transfected pHBV1.3 and derived plasmid expressing CpP25A (**A** and **C**) or CpI105T (**B** and **D**) at a range of different molar ratios. At 6 h post-transfection, the cells were mock-treated or treated with a serial concentration of AB-506 (**A** and **B**) or GLS4 (**C** and **D**) for 66 h. Cytoplasmic capsid-associated HBV DNA were quantified by a qPCR assay and plotted as the percentage of that in mock-treated control cells. EC_50_ values of the CAMs to each of the WT and mutant Cp-expressing replicon ratios are calculated from the dose-response curves of three biological replicates using Prism GraphPad version 9.
(TIF)

S2 Fig GLS4 and AB-506 accelerate the assembly of wild-type Cp149 dimers as well as WT Cp149 and Cp149-T33N dimers in 1:1 mixture. The *in vitro* Cp dimer assembly reactions in the presence of the indicated concentration of GLS4 (A) or AB-506 (B) were performed in 300 mM NaCl at 23°C for 300s and monitored by 90° light scattering using a HORIBA FluoroMax Plus.
(TIF)

S3 Fig Effects of GLS4 on HBeAg secretion in HepG2 cells. **(A)** HepG2 cells were transfected with pXF3H-P25HA_Δ_CTD-WT, pXF3H-P25HA_Δ_CTD-T33N or their combination at a molar ratio of 1:1. At 6 h post transfection, the cells were cultured with a serial concentration of GLS4. Culture media were harvested at 48 h post transfection. Intracellular p17 was detected by Western blotting using anti-HA antibody. β-actin served as a loading control. A particle gel assay was performed to detect the assembly products of p17 with HBV capsids in the cytoplasmic lysate of HepG2 cells transfected with pCMV-HBc as a positive control. (**B)** The secreted HBeAg was measured by ELISA-CLIA kit. EC_50_ values of GLS4 on HBeAg secretion from the transfected HepG2 cells were calculated from an experiment with three biological replicates by using Prism GraphPad version 9.
(TIF)

S4 Fig GLS4 treatment does not apparently alter the intracellular localization of p17. Huh7 cells transfected with p25HA_△_CTD-WT, p25HA_△_CTD-T33N or a mixture of p25HA_△_CTD-WT and p25HA_△_CTD-T33N at a molar ratio of 1:1. At 6 h post transfection, the cells were mock-treated with control solvent (DMSO) or GLS4 (10 µM) for 48 h. Intracellular p17 were visualized by immunofluorescent staining with anti-HA antibody as the first antibody and Alexa Fluor 488 conjugated goat anti-rabbit secondary antibody as the secondary antibody. Nuclei were visualized by DAPI staining. Images were taken with Nikon A1R-STED using a 60 × objective. Scale bar: 20 μm.
(TIF)

## References

[1] WHO. Hepatitis B fact sheet. 2024. https://wwwwhoint/news-room/fact-sheets/detail/hepatitis-b

[2] Gill US, Peppa D, Micco L, Singh HD, Carey I, Foster GR, et al. Interferon alpha induces sustained changes in NK cell responsiveness to Hepatitis B viral load suppression in vivo. PLoS Pathog. 2016;12(8):e1005788. doi: 10.1371/journal.ppat.1005788 27487232 PMC4972354

[3] Nishio A, Bolte FJ, Takeda K, Park N, Yu Z-X, Park H, et al. Clearance of pegylated interferon by Kupffer cells limits NK cell activation and therapy response of patients with HBV infection. Sci Transl Med. 2021;13(587):eaba6322. doi: 10.1126/scitranslmed.aba6322 33790025 PMC12109059

[4] Liu F, Campagna M, Qi Y, Zhao X, Guo F, Xu C, et al. Alpha-interferon suppresses hepadnavirus transcription by altering epigenetic modification of cccDNA minichromosomes. PLoS Pathog. 2013;9(9):e1003613. doi: 10.1371/journal.ppat.1003613 24068929 PMC3771898

[5] Sun Y, Wu X, Zhou J, Meng T, Wang B, Chen S, et al. Persistent low level of Hepatitis B virus promotes fibrosis progression during therapy. Clin Gastroenterol Hepatol. 2020;18(11):2582-2591.e6. doi: 10.1016/j.cgh.2020.03.001 32147592

[6] Dienstag JL. Benefits and risks of nucleoside analog therapy for Hepatitis B. Hepatology. 2009;49(5 Suppl):S112-21. doi: 10.1002/hep.22920 19399795

[7] Liang TJ, Block TM, McMahon BJ, Ghany MG, Urban S, Guo J-T, et al. Present and future therapies of hepatitis B: from discovery to cure. Hepatology. 2015;62(6):1893–908. doi: 10.1002/hep.28025 26239691 PMC4681668

[8] Block TM, Gish R, Guo H, Mehta A, Cuconati A, Thomas London W, et al. Chronic hepatitis B: what should be the goal for new therapies?. Antiviral Res. 2013;98(1):27–34. doi: 10.1016/j.antiviral.2013.01.006 23391846 PMC3627746

[9] Zhao Q, Liu H, Tang L, Wang F, Tolufashe G, Chang J, et al. Mechanism of interferon alpha therapy for chronic hepatitis B and potential approaches to improve its therapeutic efficacy. Antiviral Res. 2024;221:105782. doi: 10.1016/j.antiviral.2023.105782 38110058

[10] Perrillo R. Benefits and risks of interferon therapy for hepatitis B. Hepatology. 2009;49(5 Suppl):S103-11. doi: 10.1002/hep.22956 19399806

[11] Tang L, Zhao Q, Wu S, Cheng J, Chang J, Guo J-T. The current status and future directions of hepatitis B antiviral drug discovery. Expert Opin Drug Discov. 2017;12(1):5–15. doi: 10.1080/17460441.2017.1255195 27797587 PMC5444906

[12] Yang S, Zeng W, Zhang J, Lu F, Chang J, Guo J-T. Restoration of a functional antiviral immune response to chronic HBV infection by reducing viral antigen load: if not sufficient, is it necessary?. Emerg Microbes Infect. 2021;10(1):1545–54. doi: 10.1080/22221751.2021.1952851 34227927 PMC8354158

[13] Chang J, Guo F, Zhao X, Guo J-T. Therapeutic strategies for a functional cure of chronic hepatitis B virus infection. Acta Pharm Sin B. 2014;4(4):248–57. doi: 10.1016/j.apsb.2014.05.002 26579392 PMC4629125

[14] Hu J, Cheng J, Tang L, Hu Z, Luo Y, Li Y, et al. Virological basis for the cure of chronic hepatitis B. ACS Infect Dis. 2019;5(5):659–74. doi: 10.1021/acsinfecdis.8b00081 29893548 PMC8026331

[15] Block TM, Guo H, Guo J-T. Molecular virology of hepatitis B virus for clinicians. Clin Liver Dis. 2007;11(4):685–706, vii. doi: 10.1016/j.cld.2007.08.002 17981225 PMC2144742

[16] Chang C, Zhou S, Ganem D, Standring DN. Phenotypic mixing between different hepadnavirus nucleocapsid proteins reveals C protein dimerization to be cis preferential. J Virol. 1994;68(8):5225–31. doi: 10.1128/JVI.68.8.5225-5231.1994 7518533 PMC236466

[17] Conway JF, Cheng N, Zlotnick A, Wingfield PT, Stahl SJ, Steven AC. Visualization of a 4-helix bundle in the hepatitis B virus capsid by cryo-electron microscopy. Nature. 1997;386(6620):91–4. doi: 10.1038/386091a0 9052787

[18] Wynne SA, Crowther RA, Leslie AG. The crystal structure of the human hepatitis B virus capsid. Mol Cell. 1999;3(6):771–80. doi: 10.1016/s1097-2765(01)80009-5 10394365

[19] Ceres P, Zlotnick A. Weak protein-protein interactions are sufficient to drive assembly of hepatitis B virus capsids. Biochemistry. 2002;41(39):11525–31. doi: 10.1021/bi0261645 12269796

[20] Hwang N, Clement JA, Guo J-T, Du Y. The HBV capsid modulators derived from sulfamoylbenzamides and benzamides: an overview of the progress of patents. Med Chem Res. 2023;32(7):1345–68. doi: 10.1007/s00044-023-03095-x

[21] Nijampatnam B, Liotta DC. Recent advances in the development of HBV capsid assembly modulators. Curr Opin Chem Biol. 2019;50:73–9. doi: 10.1016/j.cbpa.2019.02.009 30952041

[22] Venkatakrishnan B, Katen SP, Francis S, Chirapu S, Finn MG, Zlotnick A. Hepatitis B virus capsids have diverse structural responses to small-molecule ligands bound to the heteroaryldihydropyrimidine pocket. J Virol. 2016;90(8):3994–4004. doi: 10.1128/JVI.03058-15 26842475 PMC4810570

[23] Katen SP, Tan Z, Chirapu SR, Finn MG, Zlotnick A. Assembly-directed antivirals differentially bind quasiequivalent pockets to modify hepatitis B virus capsid tertiary and quaternary structure. Structure. 2013;21(8):1406–16. doi: 10.1016/j.str.2013.06.013 23871485 PMC3756818

[24] Schlicksup CJ, Laughlin P, Dunkelbarger S, Wang JC-Y, Zlotnick A. Local stabilization of subunit-subunit contacts causes global destabilization of hepatitis B virus capsids. ACS Chem Biol. 2020;15(6):1708–17. doi: 10.1021/acschembio.0c00320 32369333 PMC8062159

[25] Klumpp K, Lam AM, Lukacs C, Vogel R, Ren S, Espiritu C, et al. High-resolution crystal structure of a hepatitis B virus replication inhibitor bound to the viral core protein. Proc Natl Acad Sci U S A. 2015;112(49):15196–201. doi: 10.1073/pnas.1513803112 26598693 PMC4679053

[26] Zhou Z, Hu T, Zhou X, Wildum S, Garcia-Alcalde F, Xu Z, et al. Heteroaryldihydropyrimidine (HAP) and Sulfamoylbenzamide (SBA) inhibit Hepatitis B virus replication by different molecular mechanisms. Sci Rep. 2017;7:42374. doi: 10.1038/srep42374 28205569 PMC5304331

[27] Zoulim F, Zlotnick A, Buchholz S, Donaldson E, Fry J, Gaggar A, et al. Nomenclature of HBV core protein-targeting antivirals. Nat Rev Gastroenterol Hepatol. 2022;19(12):748–50. doi: 10.1038/s41575-022-00700-z 36207612 PMC10442071

[28] Campagna MR, Liu F, Mao R, Mills C, Cai D, Guo F, et al. Sulfamoylbenzamide derivatives inhibit the assembly of hepatitis B virus nucleocapsids. J Virol. 2013;87(12):6931–42. doi: 10.1128/JVI.00582-13 23576513 PMC3676120

[29] Xu C, Guo H, Pan X-B, Mao R, Yu W, Xu X, et al. Interferons accelerate decay of replication-competent nucleocapsids of hepatitis B virus. J Virol. 2010;84(18):9332–40. doi: 10.1128/JVI.00918-10 20610715 PMC2937652

[30] Wu S, Zhao Q, Zhang P, Kulp J, Hu L, Hwang N, et al. Discovery and mechanistic study of benzamide derivatives that modulate hepatitis B virus capsid assembly. J Virol. 2017;91(16):e00519-17. doi: 10.1128/JVI.00519-17 28566379 PMC5533917

[31] Wu S, Luo Y, Viswanathan U, Kulp J, Cheng J, Hu Z, et al. CpAMs induce assembly of HBV capsids with altered electrophoresis mobility: implications for mechanism of inhibiting pgRNA packaging. Antiviral Res. 2018;159:1–12. doi: 10.1016/j.antiviral.2018.09.001 30201396 PMC8034245

[32] Luo Y, Cheng J, Hu Z, Ban H, Wu S, Hwang N, et al. Identification of hepatitis B virus core protein residues critical for capsid assembly, pgRNA encapsidation and resistance to capsid assembly modulators. Antiviral Res. 2021;191:105080. doi: 10.1016/j.antiviral.2021.105080 33933516

[33] Liu H, Cheng J, Viswanathan U, Chang J, Lu F, Guo J-T. Amino acid residues at core protein dimer-dimer interface modulate multiple steps of hepatitis B virus replication and HBeAg biogenesis. PLoS Pathog. 2021;17(11):e1010057. doi: 10.1371/journal.ppat.1010057 34752483 PMC8604296

[34] Tan Z, Pionek K, Unchwaniwala N, Maguire ML, Loeb DD, Zlotnick A. The interface between hepatitis B virus capsid proteins affects self-assembly, pregenomic RNA packaging, and reverse transcription. J Virol. 2015;89(6):3275–84. doi: 10.1128/JVI.03545-14 25568211 PMC4337549

[35] Cui X, Luckenbaugh L, Bruss V, Hu J. Alteration of mature nucleocapsid and enhancement of covalently closed circular DNA formation by hepatitis b virus core mutants defective in complete-virion formation. J Virol. 2015;89(19):10064–72. doi: 10.1128/JVI.01481-15 26202253 PMC4577893

[36] Huang Q, Cai D, Yan R, Li L, Zong Y, Guo L, et al. Preclinical profile and characterization of the hepatitis B virus core protein inhibitor ABI-H0731. Antimicrob Agents Chemother. 2020;64(11):e01463-20. doi: 10.1128/AAC.01463-20 32868329 PMC7577125

[37] Mani N, Cole AG, Phelps JR, Ardzinski A, Burns R, Chiu T, et al. Preclinical characterization of AB-506, an inhibitor of HBV replication targeting the viral core protein. Antiviral Res. 2022;197:105211. doi: 10.1016/j.antiviral.2021.105211 34826506

[38] Ruan L, Hadden JA, Zlotnick A. Assembly properties of hepatitis B virus core protein mutants correlate with their resistance to assembly-directed antivirals. J Virol. 2018;92(20):e01082-18. doi: 10.1128/JVI.01082-18 30089690 PMC6158430

[39] Bassit L, Amblard F, Patel D, Biteau N, Chen Z, Kasthuri M, et al. The premise of capsid assembly modulators towards eliminating HBV persistence. Expert Opin Drug Discov. 2023;18(9):1031–41. doi: 10.1080/17460441.2023.2239701 37477111 PMC10530454

[40] Viswanathan U, Mani N, Hu Z, Ban H, Du Y, Hu J, et al. Targeting the multifunctional HBV core protein as a potential cure for chronic hepatitis B. Antiviral Res. 2020;182:104917. doi: 10.1016/j.antiviral.2020.104917 32818519 PMC8050868

[41] Yuen M-F, Berliba E, Sukeepaisarnjaroen W, Ahn SH, Tanwandee T, Lim Y-S, et al. Safety, pharmacokinetics, and antiviral activity of the capsid inhibitor AB-506 from Phase 1 studies in healthy subjects and those with hepatitis B. Hepatol Commun. 2022;6(12):3457–72. doi: 10.1002/hep4.2095 36194181 PMC9701477

[42] Verbinnen T, Talloen W, Janssen HLA, Zoulim F, Shukla U, Vandenbossche JJ, et al. Viral sequence analysis of chronic hepatitis B patients treated with the capsid assembly modulator JNJ-56136379 in the JADE phase 2a study. Antiviral Res. 2023;216:105660. doi: 10.1016/j.antiviral.2023.105660 37385475

[43] Janssen HLA, Hou J, Asselah T, Chan HLY, Zoulim F, Tanaka Y, et al. Randomised phase 2 study (JADE) of the HBV capsid assembly modulator JNJ-56136379 with or without a nucleos(t)ide analogue in patients with chronic hepatitis B infection. Gut. 2023;72(7):1385–98. doi: 10.1136/gutjnl-2022-328041 36697207 PMC10313999

[44] Tolufashe G, Viswanathan U, Kulp J, Guo J-T. Computational approaches to predict hepatitis B virus capsid protein mutations that confer resistance to capsid assembly modulators. Viruses. 2025;17(3):332. doi: 10.3390/v17030332 40143261 PMC11945318

[45] Yu Y, Kass MA, Zhang M, Youssef N, Freije CA, Brock KP, et al. Deep mutational scanning of hepatitis B virus reveals a mechanism for cis-preferential reverse transcription. Cell. 2024;187(11):2735-2745.e12. doi: 10.1016/j.cell.2024.04.008 38723628 PMC11127778

[46] Bourne CR, Katen SP, Fulz MR, Packianathan C, Zlotnick A. A mutant hepatitis B virus core protein mimics inhibitors of icosahedral capsid self-assembly. Biochemistry. 2009;48(8):1736–42. doi: 10.1021/bi801814y 19196007 PMC2880625

[47] Zhao Q, Chang J, Rijnbrand R, Lam AM, Sofia MJ, Cuconati A, et al. Pregenomic RNA launch hepatitis B virus replication system facilitates the mechanistic study of antiviral agents and drug-resistant variants on covalently closed circular DNA synthesis. J Virol. 2022;96(24):e0115022. doi: 10.1128/jvi.01150-22 36448800 PMC9769369

[48] Yu Y, Schneider WM, Kass MA, Michailidis E, Acevedo A, Pamplona Mosimann AL, et al. An RNA-based system to study hepatitis B virus replication and evaluate antivirals. Sci Adv. 2023;9(15):eadg6265. doi: 10.1126/sciadv.adg6265 37043562 PMC10096565

[49] Lin J, Yin L, Xu X-Z, Sun H-C, Huang Z-H, Ni X-Y, et al. Bay41-4109-induced aberrant polymers of hepatitis b capsid proteins are removed via STUB1-promoted p62-mediated macroautophagy. PLoS Pathog. 2022;18(1):e1010204. doi: 10.1371/journal.ppat.1010204 35030230 PMC8824320

[50] Kornyeyev D, Song Z, Eng S, Soulette C, Ramirez R, Tang J, et al. Selective depletion of HBV-infected hepatocytes by class A capsid assembly modulators requires high levels of intrahepatic HBV core protein. Antimicrob Agents Chemother. 2024;68(7):e0042024. doi: 10.1128/aac.00420-24 38780261 PMC11232385

[51] Kum DB, Vanrusselt H, Acosta Sanchez A, Taverniti V, Verrier ER, Baumert TF, et al. Class A capsid assembly modulator RG7907 clears HBV-infected hepatocytes through core-dependent hepatocyte death and proliferation. Hepatology. 2023;78(4):1252–65. doi: 10.1097/HEP.0000000000000428 37102495

[52] Vanrusselt H, Kum DB, Taverniti V, Liu C, Acosta Sanchez A, Corthout N, et al. Novel non-HAP class A HBV capsid assembly modulators have distinct in vitro and in vivo profiles. J Virol. 2023;97(10):e0072223. doi: 10.1128/jvi.00722-23 37754761 PMC10617565

[53] Berke JM, Tan Y, Sauviller S, Wu D-T, Zhang K, Conceição-Neto N, et al. Class A capsid assembly modulator apoptotic elimination of hepatocytes with high HBV core antigen level in vivo is dependent on de novo core protein translation. J Virol. 2024;98(3):e0150223. doi: 10.1128/jvi.01502-23 38315015 PMC10949496

[54] Taverniti V, Meiss-Heydmann L, Gadenne C, Vanrusselt H, Kum DB, Giannone F, et al. CAM-A-dependent HBV core aggregation induces apoptosis through ANXA1. JHEP Rep. 2024;6(10):101134. doi: 10.1016/j.jhepr.2024.101134 39386256 PMC11462251

[55] Bourne CR, Finn MG, Zlotnick A. Global structural changes in hepatitis B virus capsids induced by the assembly effector HAP1. J Virol. 2006;80(22):11055–61. doi: 10.1128/JVI.00933-06 16943288 PMC1642186

[56] Guo F, Zhao Q, Sheraz M, Cheng J, Qi Y, Su Q, et al. HBV core protein allosteric modulators differentially alter cccDNA biosynthesis from de novo infection and intracellular amplification pathways. PLoS Pathog. 2017;13(9):e1006658. doi: 10.1371/journal.ppat.1006658 28945802 PMC5629035

[57] Berke JM, Dehertogh P, Vergauwen K, Mostmans W, Vandyck K, Raboisson P, et al. Antiviral properties and mechanism of action studies of the hepatitis B virus capsid assembly modulator JNJ-56136379. Antimicrob Agents Chemother. 2020;64(5):e02439-19. doi: 10.1128/AAC.02439-19 32094138 PMC7179615

[58] Hu J, Tang L, Cheng J, Zhou T, Li Y, Chang J, et al. Hepatitis B virus nucleocapsid uncoating: biological consequences and regulation by cellular nucleases. Emerg Microbes Infect. 2021;10(1):852–64. doi: 10.1080/22221751.2021.1919034 33870849 PMC8812769

[59] Cai D, Nie H, Yan R, Guo J-T, Block TM, Guo H. A southern blot assay for detection of hepatitis B virus covalently closed circular DNA from cell cultures. Methods Mol Biol. 2013;1030:151–61. doi: 10.1007/978-1-62703-484-5_13 23821267 PMC5060941

[60] Yan Z, Wu D, Hu H, Zeng J, Yu X, Xu Z, et al. Direct inhibition of hepatitis B e antigen by core protein allosteric modulator. Hepatology. 2019;70(1):11–24. doi: 10.1002/hep.30514 30664279 PMC6618080

[61] Watts NR, Palmer IW, Eren E, Steven AC, Wingfield PT. Capsids of hepatitis B virus e antigen with authentic C termini are stabilized by electrostatic interactions. FEBS Lett. 2020;594(6):1052–61. doi: 10.1002/1873-3468.13706 31792961 PMC7337981

[62] Lutomski CA, Lyktey NA, Pierson EE, Zhao Z, Zlotnick A, Jarrold MF. Multiple pathways in capsid assembly. J Am Chem Soc. 2018;140(17):5784–90. doi: 10.1021/jacs.8b01804 29672035 PMC6347577

[63] Zlotnick A, Venkatakrishnan B, Tan Z, Lewellyn E, Turner W, Francis S. Core protein: a pleiotropic keystone in the HBV lifecycle. Antiviral Res. 2015;121:82–93. doi: 10.1016/j.antiviral.2015.06.020 26129969 PMC4537649

[64] Stray SJ, Zlotnick A. BAY 41-4109 has multiple effects on Hepatitis B virus capsid assembly. J Mol Recognit. 2006;19(6):542–8. doi: 10.1002/jmr.801 17006877

[65] Katen SP, Chirapu SR, Finn MG, Zlotnick A. Trapping of hepatitis B virus capsid assembly intermediates by phenylpropenamide assembly accelerators. ACS Chem Biol. 2010;5(12):1125–36. doi: 10.1021/cb100275b 20845949 PMC3003741

[66] Hu Z, Ban H, Zheng H, Liu M, Chang J, Guo J-T. Protein phosphatase 1 catalyzes HBV core protein dephosphorylation and is co-packaged with viral pregenomic RNA into nucleocapsids. PLoS Pathog. 2020;16(7):e1008669. doi: 10.1371/journal.ppat.1008669 32702076 PMC7402523

[67] Zlotnick A, Cheng N, Conway JF, Booy FP, Steven AC, Stahl SJ, et al. Dimorphism of hepatitis B virus capsids is strongly influenced by the C-terminus of the capsid protein. Biochemistry. 1996;35(23):7412–21. doi: 10.1021/bi9604800 8652518

[68] Sun X, Li D, Wang Z, Liu Q, Wei Y, Liu T. A dimorphism shift of hepatitis B virus capsids in response to ionic conditions. Nanoscale. 2018;10(36):16984–9. doi: 10.1039/c8nr03370f 30183040

[69] Ning X, Nguyen D, Mentzer L, Adams C, Lee H, Ashley R, et al. Secretion of genome-free hepatitis B virus--single strand blocking model for virion morphogenesis of para-retrovirus. PLoS Pathog. 2011;7(9):e1002255. doi: 10.1371/journal.ppat.1002255 21966269 PMC3178560

[70] Zhao Q, Hu Z, Cheng J, Wu S, Luo Y, Chang J, et al. Hepatitis B virus core protein dephosphorylation occurs during pregenomic RNA encapsidation. J Virol. 2018;92(13):e02139-17. doi: 10.1128/JVI.02139-17 29669831 PMC6002726

[71] Ning X, Luckenbaugh L, Liu K, Bruss V, Sureau C, Hu J. Common and distinct capsid and surface protein requirements for secretion of complete and genome-free hepatitis B virions. J Virol. 2018;92(14):e00272-18. doi: 10.1128/JVI.00272-18 29743374 PMC6026761

[72] Tong S, Kim KH, Chante C, Wands J, Li J. Hepatitis B virus e antigen variants. Int J Med Sci. 2005;2(1):2–7. 15968333 10.7150/ijms.2.2 PMC1142218

[73] Milich DR. Is the function of the HBeAg really unknown?. Hum Vaccin Immunother. 2019;15(9):2187–91. doi: 10.1080/21645515.2019.1607132 31063442 PMC6773382

[74] Lau DTY, Ganova-Raeva L, Wang J, Mogul D, Chung RT, Lisker-Melman M, et al. Precore and basal core promoter hepatitis B virus (HBV) variants are present from a young age and differ across HBV genotypes. Hepatology. 2021;73(5):1637–51. doi: 10.1002/hep.31506 32860463 PMC8570313

[75] Lahlali T, Berke JM, Vergauwen K, Foca A, Vandyck K, Pauwels F, et al. Novel potent capsid assembly modulators regulate multiple steps of the hepatitis B virus life cycle. Antimicrob Agents Chemother. 2018;62(10):e00835-18. doi: 10.1128/AAC.00835-18 30012770 PMC6153789

[76] Guo H, Jiang D, Zhou T, Cuconati A, Block TM, Guo J-T. Characterization of the intracellular deproteinized relaxed circular DNA of hepatitis B virus: an intermediate of covalently closed circular DNA formation. J Virol. 2007;81(22):12472–84. doi: 10.1128/JVI.01123-07 17804499 PMC2169032

[77] Sussman NL, Chong MG, Koussayer T, He DE, Shang TA, Whisennand HH, et al. Reversal of fulminant hepatic failure using an extracorporeal liver assist device. Hepatology. 1992;16(1):60–5. doi: 10.1002/hep.1840160112 1618484

[78] Guo F, Tang L, Shu S, Sehgal M, Sheraz M, Liu B, et al. Activation of stimulator of interferon genes in hepatocytes suppresses the replication of hepatitis B virus. Antimicrob Agents Chemother. 2017;61(10):e00771-17. doi: 10.1128/AAC.00771-17 28717041 PMC5610522

[79] Blight KJ, McKeating JA, Rice CM. Highly permissive cell lines for subgenomic and genomic hepatitis C virus RNA replication. J Virol. 2002;76(24):13001–14. doi: 10.1128/jvi.76.24.13001-13014.2002 12438626 PMC136668

[80] Mao R, Zhang J, Jiang D, Cai D, Levy JM, Cuconati A. Indoleamine 2,3-dioxygenase mediates the antiviral effect of gamma interferon against hepatitis B virus in human hepatocyte-derived cells. J Virol. 2011;85(2):1048–57. doi: JVI.01998-10 21084489 10.1128/JVI.01998-10 PMC3019998

[81] Guo H, Zhou T, Jiang D, Cuconati A, Xiao G-H, Block TM, et al. Regulation of hepatitis B virus replication by the phosphatidylinositol 3-kinase-akt signal transduction pathway. J Virol. 2007;81(18):10072–80. doi: 10.1128/JVI.00541-07 17609269 PMC2045390

[82] Yang S, Shen Z, Kang Y, Sun L, Viswanathan U, Guo H, et al. A putative amphipathic alpha helix in hepatitis B virus small envelope protein plays a critical role in the morphogenesis of subviral particles. J Virol. 2021;95(8):e02399-20. doi: 10.1128/JVI.02399-20 33536177 PMC8103704

[83] Zlotnick A, Lee A, Bourne CR, Johnson JM, Domanico PL, Stray SJ. In vitro screening for molecules that affect virus capsid assembly (and other protein association reactions). Nat Protoc. 2007;2(3):490–8. doi: 10.1038/nprot.2007.60 17406612 PMC2099249

[84] Wingfield PT, Stahl SJ, Williams RW, Steven AC. Hepatitis core antigen produced in Escherichia coli: subunit composition, conformational analysis, and in vitro capsid assembly. Biochemistry. 1995;34(15):4919–32. doi: 10.1021/bi00015a003 7711014

[85] Ruan L, Hadden JA, Zlotnick A. Assembly properties of hepatitis B virus core protein mutants correlate with their resistance to assembly-directed antivirals. J Virol. 2018;92(20):e01082-18. doi: 10.1128/JVI.01082-18 30089690 PMC6158430

[86] Starr CA, Nair S, Huang S-Y, Hagan MF, Jacobson SC, Zlotnick A. Engineering metastability into a virus-like particle to enable triggered dissociation. J Am Chem Soc. 2023;145(4):2322–31. doi: 10.1021/jacs.2c10937 36651799 PMC10018796

[87] Liu Y, Liu H, Hu Z, Ding Y, Pan X-B, Zou J, et al. Hepatitis B virus virions produced under nucleos(t)ide analogue treatment are mainly not infectious because of irreversible DNA chain termination. Hepatology. 2020;71(2):463–76. doi: 10.1002/hep.30844 31278760 PMC7028043

[88] Xia Y, Stadler D, Ko C, Protzer U. Analyses of HBV cccDNA quantification and modification. Methods Mol Biol. 2017;1540:59–72. doi: 10.1007/978-1-4939-6700-1_6 27975308

