# Supplementary figures and images for: HBV capsid assembly modulators differentially modulate the assembly of wild-type and drug-resistant core protein chimeric nucleocapsids and empty capsids
Source: PLoS Pathog. 2025 Aug 5;21(8):e1013391. doi: 10.1371/journal.ppat.1013391 (PMC12342251; doi:10.1371/journal.ppat.1013391)

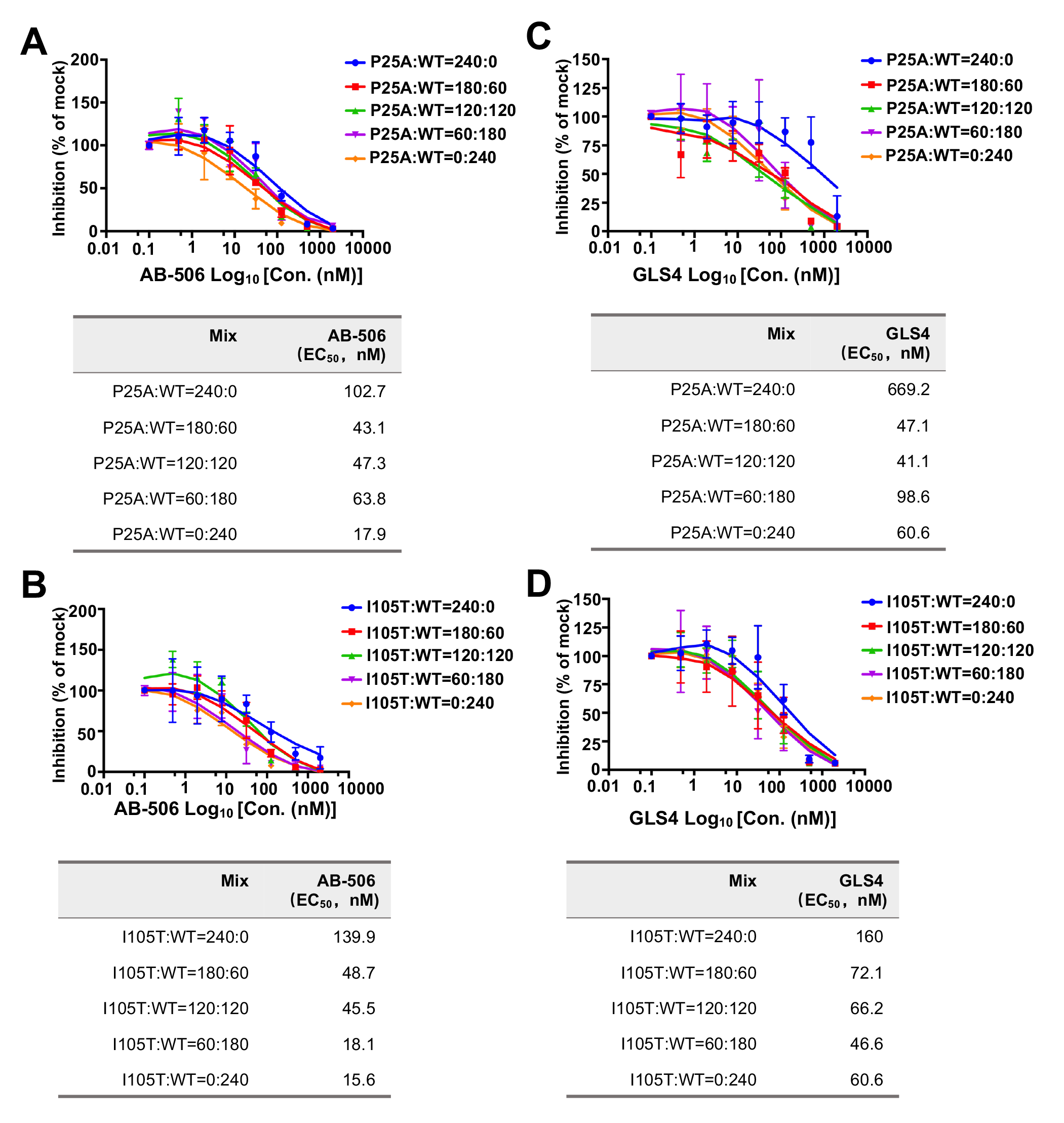

Supplement: S1 Fig — HepG2 cells were co-transfected pHBV1.3 and derived plasmid expressing CpP25A (A and C) or CpI105T (B and D) at a range of different molar ratios. At 6 h post-transfection, the cells were mock-treated or treated with a serial concentration of AB-506 (A and B) or GLS4 (C and D) for 66 h. Cytoplasmic capsid-associated HBV DNA were quantified by a qPCR assay and plotted as the percentage of that in mock-treated control cells. EC50 values of the CAMs to each of the WT and mutant Cp-expressing replicon ratios are calculated from the dose-response curves of three biological replicates using Prism GraphPad version 9. (TIF) [file ppat.1013391.s001.tif]

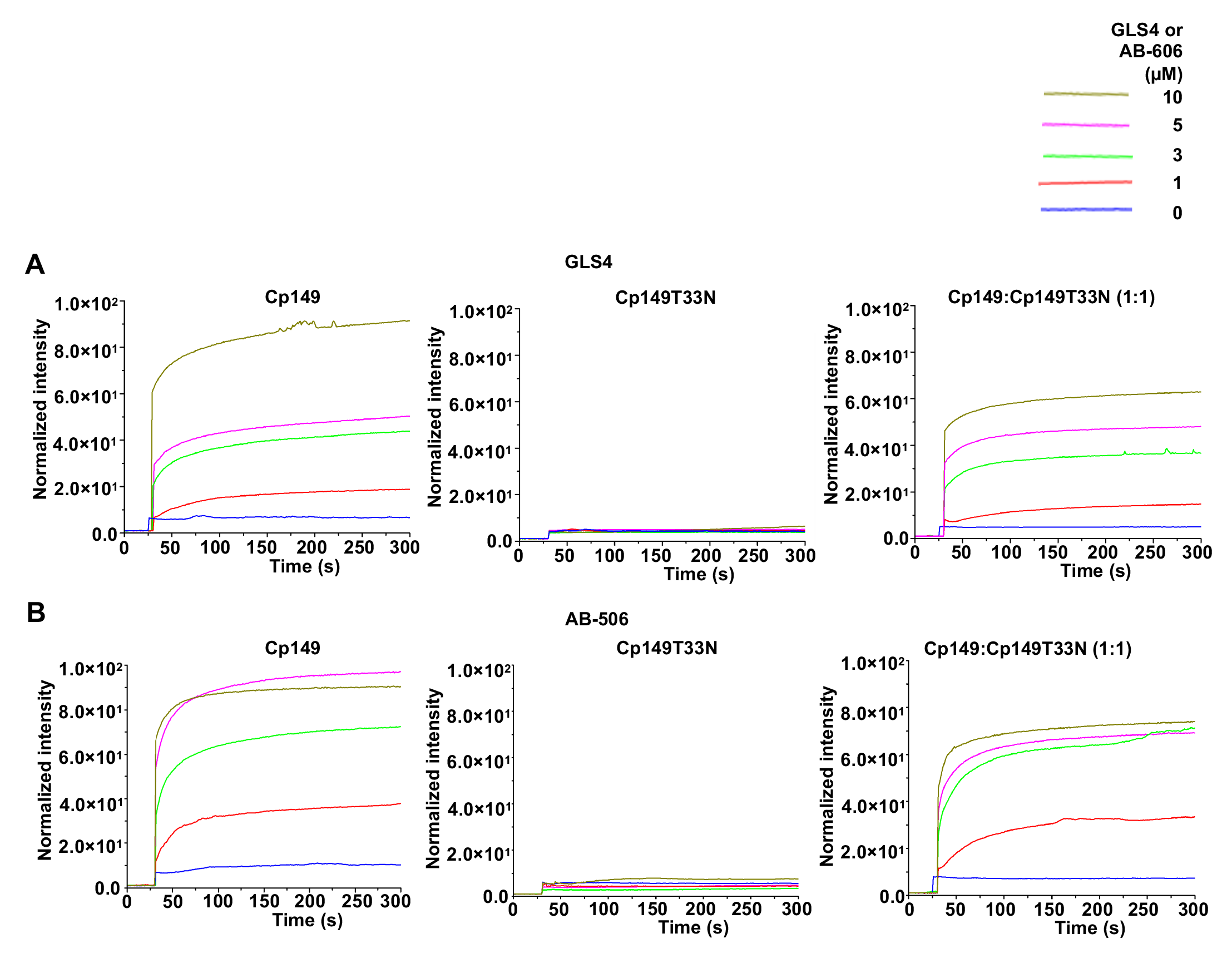

Supplement: S2 Fig — The in vitro Cp dimer assembly reactions in the presence of the indicated concentration of GLS4 (A) or AB-506 (B) were performed in 300 mM NaCl at 23°C for 300s and monitored by 90° light scattering using a HORIBA FluoroMax Plus. (TIF) [file ppat.1013391.s002.tif]

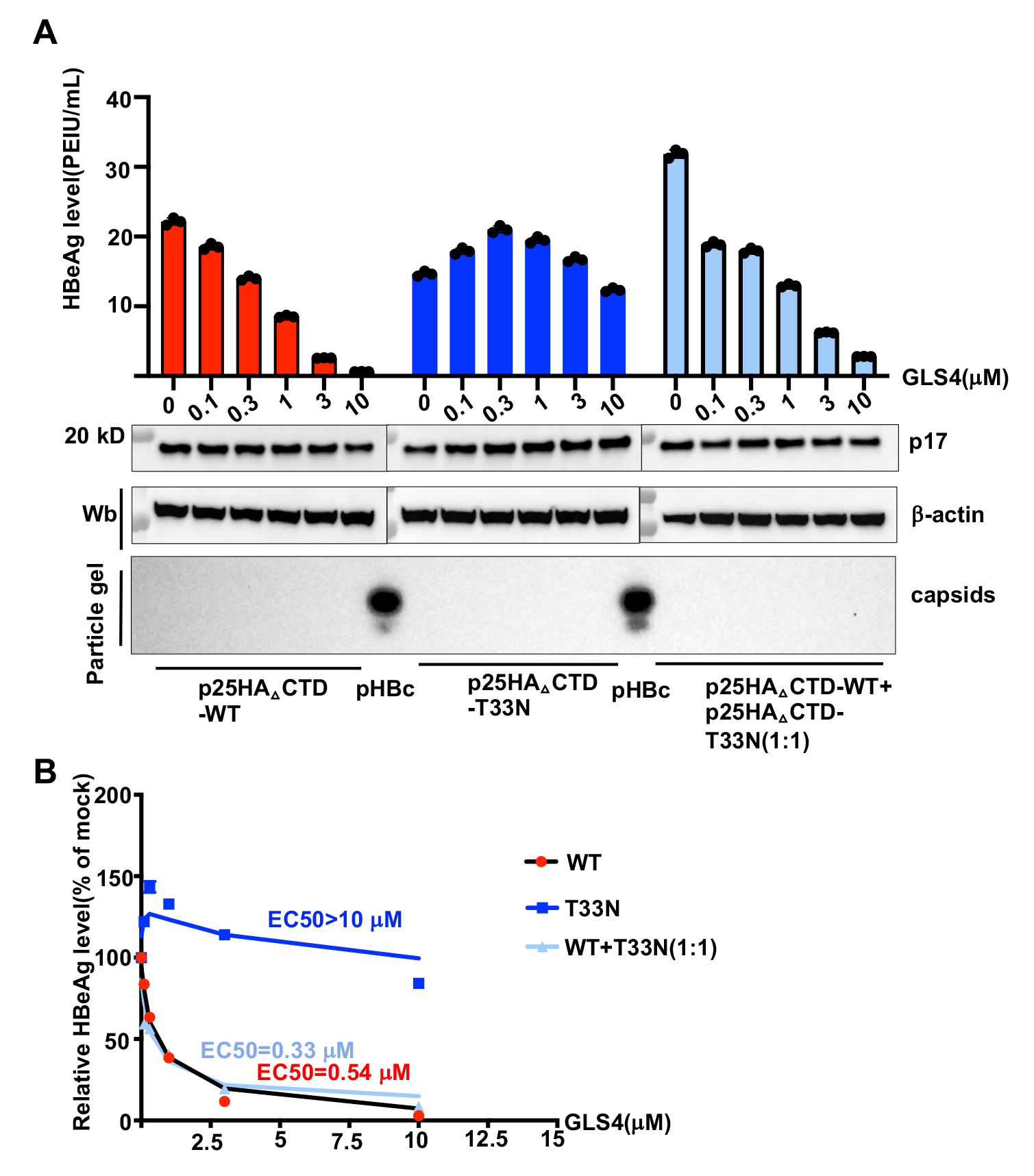

Supplement: S3 Fig — (A) HepG2 cells were transfected with pXF3H-P25HAΔCTD-WT, pXF3H-P25HAΔCTD-T33N or their combination at a molar ratio of 1:1. At 6 h post transfection, the cells were cultured with a serial concentration of GLS4. Culture media were harvested at 48 h post transfection. Intracellular p17 was detected by Western blotting using anti-HA antibody. β-actin served as a loading control. A particle gel assay was performed to detect the assembly products of p17 with HBV capsids in the cytoplasmic lysate of HepG2 cells transfected with pCMV-HBc as a positive control. (B) The secreted HBeAg was measured by ELISA-CLIA kit. EC50 values of GLS4 on HBeAg secretion from the transfected HepG2 cells were calculated from an experiment with three biological replicates by using Prism GraphPad version 9. (TIF) [file ppat.1013391.s003.tif]

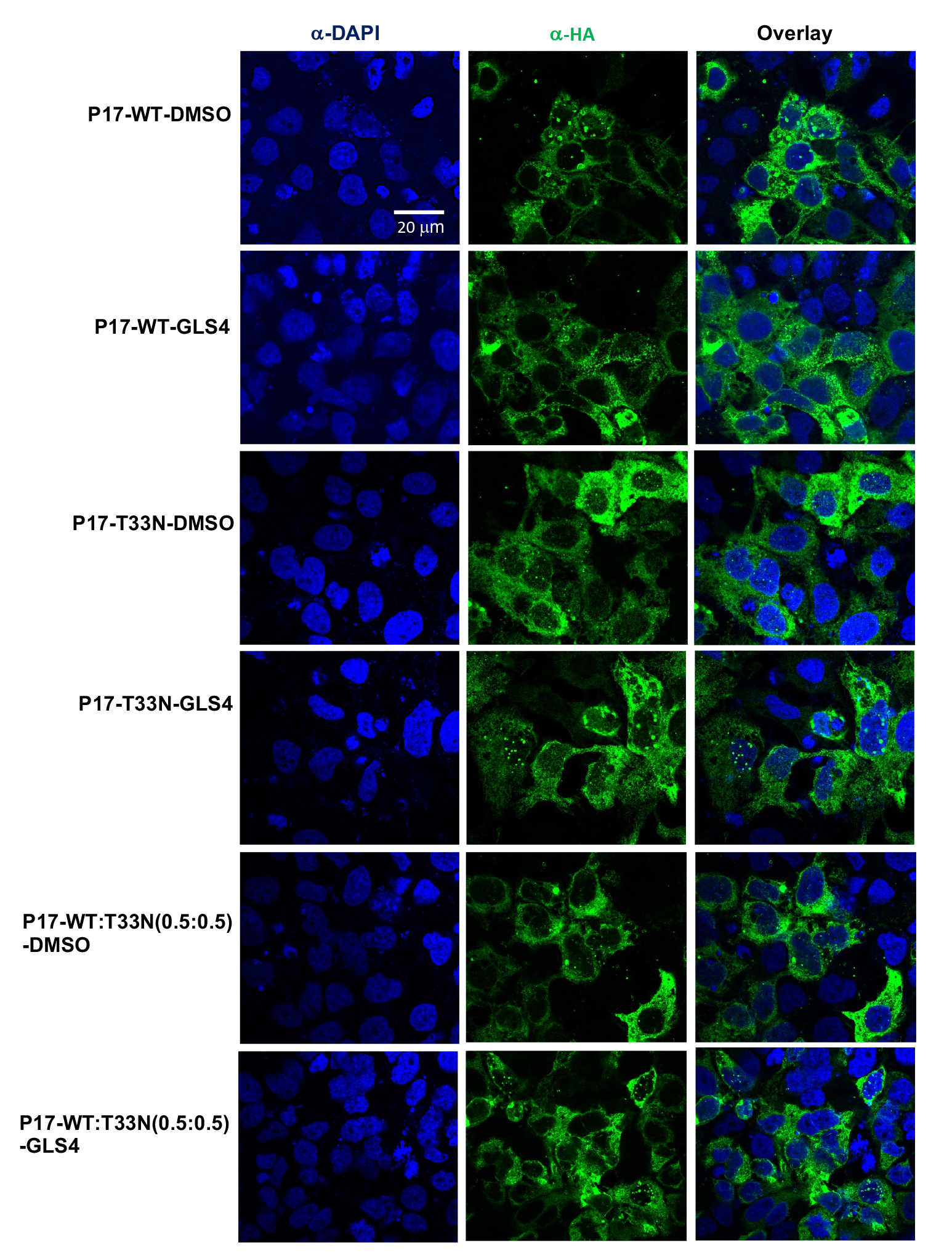

Supplement: S4 Fig — Huh7 cells transfected with p25HA△CTD-WT, p25HA△CTD-T33N or a mixture of p25HA△CTD-WT and p25HA△CTD-T33N at a molar ratio of 1:1. At 6 h post transfection, the cells were mock-treated with control solvent (DMSO) or GLS4 (10 µM) for 48 h. Intracellular p17 were visualized by immunofluorescent staining with anti-HA antibody as the first antibody and Alexa Fluor 488 conjugated goat anti-rabbit secondary antibody as the secondary antibody. Nuclei were visualized by DAPI staining. Images were taken with Nikon A1R-STED using a 60 × objective. Scale bar: 20 μm. (TIF) [file ppat.1013391.s004.tif]
